# Strategic Thinking and Entrepreneurial Orientation as Drivers of Adaptive Resilience in Global Manufacturing SMEs: A Narrative Review of Dynamic Capabilities and Cross-Context Variations

**DOI:** 10.12688/f1000research.178316.1

**Published:** 2026-03-03

**Authors:** Tom Ongesa Nyamboga

**Affiliations:** 1 business administration, Kampala International University - Western Campus, Bushenyi, Western Region, Uganda

**Keywords:** Strategic thinking, Entrepreneurial orientation, Adaptive resilience, Organisational resilience, Manufacturing SMEs

## Abstract

In this review we investigate how strategic thinking and entrepreneurial orientation jointly foster adaptive resilience in manufacturing SMEs, addressing a critical knowledge gap regarding their integrated role across sensing, seizing, reconfiguring, learning, and strategic renewal capabilities in diverse institutional contexts. Our review aims to synthesise recent evidence on mechanisms of adaptive resilience, identify persistent challenges, and inform both theory and policy. Anchored in Dynamic Capabilities Theory and Organisational Resilience Theory, the review employs a qualitative thematic analysis of literature from major electronic databases spanning 2020 to 2025, focusing on empirical and conceptual studies of global manufacturing SMEs. Our findings indicate that strategic thinking and entrepreneurial orientation enable early recognition of environmental signals, disciplined opportunity prioritisation, resource reconfiguration, experiential learning, and continuous strategic renewal. Persistent challenges comprise resource constraints in emerging economies, institutional barriers, variability in strategic adoption, and limited longitudinal evidence on resilience outcomes. The review concludes that adaptive resilience is a dynamic, context-sensitive process requiring deliberate strategic design and proactive entrepreneurial action. Its contribution is twofold: empirically, by consolidating contemporary insights into SME resilience mechanisms, and theoretically, by extending dynamic capabilities and organisational resilience frameworks to complex, small-scale manufacturing contexts. For policymakers, the findings underscore the importance of creating enabling ecosystems, targeted innovation financing, capability development programmes, and context-specific interventions to strengthen SME resilience, competitiveness, and long-term strategic adaptability.

## Introduction

Manufacturing SMEs constitute a critical pillar of global industrial systems through employment creation, technological diffusion, and participation in domestic and international value chains. ^1, 2^ Across both advanced and emerging economies, manufacturing SMEs operate in environments characterised by rapid technological change, volatile input markets, supply chain disruptions, regulatory shifts, and intensifying competitive pressure. These conditions have heightened exposure to recurrent shocks and prolonged uncertainty, positioning organisational resilience as a central concern for SME sustainability rather than a peripheral crisis response. ^3, 4^

Recent scholarship increasingly conceptualises resilience as an adaptive capability that enables firms not only to withstand disruption but also to reconfigure strategies, processes, and resources in anticipation of future uncertainty. ^5^ Within this framing, cognitive and behavioural dimensions of strategy emerge as essential enablers of adaptive resilience. Strategic thinking equips SME leaders with the capacity to interpret complex environments, integrate long-term perspectives with immediate operational decisions, and align organisational actions with evolving external conditions. ^6, 7^ Entrepreneurial orientation complements this cognitive capability by shaping firm-level behaviours such as innovativeness, proactiveness, and calculated risk-taking that support opportunity recognition and strategic renewal. ^8^

Studies in strategic management and entrepreneurship have separately demonstrated the relevance of strategic thinking and entrepreneurial orientation for performance, innovation, and competitiveness in manufacturing SMEs. ^9^ Parallel streams of research on organisational resilience have focused on recovery, robustness, and continuity, particularly in response to systemic disruptions. ^10– 12^ However, limited effort has been made to synthesise these strands to explain how strategic thinking and entrepreneurial orientation jointly contribute to adaptive resilience. Even fewer studies adopt a dynamic capabilities perspective to explain how these strategic attributes translate into sustained organisational adaptation across diverse institutional and market contexts.

This narrative review addresses this gap by integrating insights from strategic thinking, entrepreneurial orientation, resilience, and dynamic capabilities literature. The review focuses on manufacturing SMEs across global contexts to illuminate cross-context variations and conceptual inconsistencies, thereby offering a coherent foundation for advancing theory and guiding future empirical research.

### Problem statement

Despite growing recognition of resilience as a strategic imperative for manufacturing SMEs, existing literature provides an incomplete and fragmented understanding of its strategic antecedents. Research commonly examines strategic thinking, entrepreneurial orientation, and organisational resilience as discrete constructs, resulting in limited insight into their interactive effects and cumulative influence on adaptive resilience. Where resilience is investigated, emphasis frequently centres on short-term recovery or operational stability, overlooking adaptive and transformative processes that are critical ^13, 14^ for long-term survival in volatile manufacturing environments.

Theoretical approaches further constrain understanding, as many studies rely on static resource-based explanations that inadequately capture the dynamic, cognitive, and behavioural mechanisms through which SMEs respond to uncertainty. Integration of dynamic capabilities theory remains limited, restricting explanation of how strategic thinking and entrepreneurial orientation enable firms to sense environmental shifts, seize emerging opportunities, and reconfigure resources over time. Empirical evidence also remains uneven across regions, with a concentration of studies in specific national or economic contexts, limiting the generalisability of findings to the global manufacturing SME landscape.

These limitations hinder the development of a unified conceptual framework capable of explaining how manufacturing SMEs build adaptive resilience through strategic thinking and entrepreneurial orientation. The absence of a systematic narrative synthesis that consolidates theoretical perspectives, empirical findings, and contextual variations constrains scholarly advancement and weakens practical guidance for SME leaders and policymakers. Addressing this gap necessitates a comprehensive narrative review that clarifies conceptual relationships, identifies knowledge gaps, and advances an integrated understanding of strategic thinking and entrepreneurial orientation as drivers of adaptive resilience in global manufacturing SMEs.

### Specific objectives of the study

• To synthesise and clarify the role of strategic thinking and entrepreneurial orientation as strategic antecedents of adaptive resilience in manufacturing SMEs.
• To examine the conceptual and empirical linkages between strategic thinking, entrepreneurial orientation, and adaptive resilience through the lens of dynamic capabilities theory.
• To analyse the mediating role of dynamic capabilities, specifically sensing, seizing, and reconfiguring, in the relationship between strategic attributes and adaptive resilience in manufacturing SMEs.
• To identify cross-context variations in the relationships among strategic thinking, entrepreneurial orientation, dynamic capabilities, and adaptive resilience across advanced and emerging economies.
• To explore the influence of institutional, market, and structural factors on the development of adaptive resilience in manufacturing SMEs across different economic contexts.
• To identify theoretical inconsistencies, methodological gaps, and underexplored areas in the existing literature to inform future empirical research and theory development.

### Rationale of the study

Manufacturing SMEs operate under heightened volatility driven by technological change, global supply chain fragility, regulatory pressures, and competitive intensity, making adaptive resilience a strategic necessity rather than an episodic response. Existing literature addresses strategic thinking, entrepreneurial orientation, and resilience largely in isolation, which limits understanding of how these constructs interact to support sustained adaptation. The dominance of static resource-based explanations further restricts insight into the cognitive and behavioural mechanisms through which SMEs navigate uncertainty over time. A narrative synthesis grounded in dynamic capabilities theory provides a more suitable lens for capturing the processes of sensing, seizing, and reconfiguring that underpin adaptive resilience. By integrating diverse theoretical strands and empirical evidence across contexts, the study responds to the need for a coherent and globally relevant explanation of resilience-building in manufacturing SMEs.

### Purpose of the study

The purpose of this study is to develop an integrated conceptual understanding of how strategic thinking and entrepreneurial orientation jointly drive adaptive resilience in global manufacturing SMEs through dynamic capabilities. Through a narrative review of extant literature, the study aims to consolidate fragmented knowledge, clarify conceptual relationships, and illuminate contextual differences that shape resilience outcomes. The study seeks to advance strategic management and entrepreneurship theory by positioning adaptive resilience as a dynamic, capability-based outcome of strategic cognition and entrepreneurial behaviour. At a practical level, the study aims to provide a theoretically grounded foundation to inform future empirical research, managerial decision-making, and policy interventions targeted at strengthening SME resilience in volatile manufacturing environments.

### Primary research question

How do strategic thinking and entrepreneurial orientation jointly contribute to adaptive resilience in global manufacturing SMEs through dynamic capabilities across different institutional and market contexts?

## Materials and methods

The review adopted an elaborate methodology as shown in Table 1.

**Table 1.** Summary of key methodological procedures used in the review.

Methodological component	Critical aspects captured
**Research Approach**	Systematic qualitative literature review examining adaptive resilience in manufacturing SMEs (2020–2025).
**Data Sources**	Peer-reviewed databases (Scopus, Web of Science, ScienceDirect, Google Scholar) and grey literature including policy briefs, institutional reports, and SME case studies.
**Data Sources**	Peer-reviewed databases (Scopus, Web of Science, ScienceDirect, Google Scholar) and grey literature including policy briefs, institutional reports, and SME case studies.
**Search Strategy**	Use of targeted keywords on resilience, strategic capabilities, and SMEs; Boolean operators and truncation applied; iterative refinement of search strings.
**Inclusion Criteria**	English studies (2020–2025) addressing strategic capabilities, entrepreneurial orientation, or resilience in manufacturing SMEs; empirical and conceptual works included.
**Exclusion Criteria**	Studies on large firms, non-manufacturing sectors, weak methodological clarity, or limited relevance excluded.
**Data Analysis Technique**	Thematic synthesis; themes aligned with dynamic capabilities and resilience perspectives.
**Rigour Measures**	Triangulation of sources, quality appraisal, consistency checks, and peer feedback to reduce bias.
**Reflexivity**	Ongoing documentation of researcher assumptions and interpretive decisions to enhance transparency and credibility.

### Data collection methods

This study employed a systematic qualitative review approach to gather evidence on adaptive resilience in manufacturing SMEs between 2020 and 2025. Data collection focused on both peer-reviewed and grey literature to ensure a comprehensive understanding of current practices and theoretical developments. Major electronic databases, including Scopus, Web of Science, ScienceDirect, and Google Scholar, were searched to identify relevant journal articles, conference proceedings, and industry reports. Grey literature, such as policy briefs, institutional reports, and case studies from SME support organisations, was incorporated to capture practical insights and contextual examples of resilience mechanisms. The collection process also involved citation tracking of key studies to identify additional relevant literature that may not have appeared in initial searches.

### Search keywords

A set of carefully constructed search keywords was employed to ensure a balance between specificity and breadth. Primary keywords included “manufacturing SMEs,” “adaptive resilience,” “strategic thinking,” “entrepreneurial orientation,” “dynamic capabilities,” “strategic renewal,” “knowledge integration,” and “resource reconfiguration.” Boolean operators such as AND, OR, and NOT were applied to refine searches, and truncation (e.g., “resilien*”) was used to capture variations in terminology. The search strategy was iteratively refined based on preliminary screening outcomes to maximise retrieval of relevant studies without overwhelming the dataset with irrelevant results.

### Screening and inclusion criteria

Screening involved a multi-stage process including title review, abstract evaluation, and full-text assessment. Studies were included if they were published between 2020 and 2025, written in English, and directly addressed the role of strategic capabilities, entrepreneurial orientation, or resilience mechanisms in manufacturing SMEs. Both empirical and conceptual studies were considered if they provided insight into how SMEs sense, seize, reconfigure, or renew resources and strategies to enhance adaptive capacity.

### Exclusion criteria

Studies were excluded if they focused solely on large enterprises, non-manufacturing sectors, or contexts unrelated to strategic and entrepreneurial capabilities. Literature lacking methodological transparency, practical relevance, or sufficient evidence to support conclusions was also excluded. Non-English studies and publications prior to 2020 were omitted to maintain temporal and linguistic consistency.

### Data analysis

Data were analysed using thematic synthesis, allowing the identification of recurring patterns, mechanisms, and contextual factors across studies. Emerging themes were mapped against the dynamic capabilities and organisational resilience frameworks to ensure conceptual coherence. NVivo software was employed to code, categorise, and visualise thematic relationships, facilitating systematic comparison across diverse studies and regional contexts.

### Evaluation and rigour enhancement

Rigour was ensured through triangulation of findings from empirical and conceptual studies, cross-validation of evidence, and iterative discussions among the research team. Quality appraisal criteria assessed methodological transparency, relevance, and robustness of evidence, while consistency checks and peer feedback were used to mitigate selection and interpretive biases.

### Reflexivity

Reflexivity was maintained throughout the review process, with conscious acknowledgment of the researcher’s assumptions, prior knowledge, and potential influence on theme identification. Reflective notes were documented to monitor interpretive decisions and ensure transparency, strengthening the credibility and validity of the findings.

### Theoretical framework

This review is anchored on Dynamic capabilities theory was pronounced by Teece, Pisano and Shuen in 1997. The theory posits that firm competitiveness in volatile and uncertain environments depends not merely on the possession of valuable resources, but on the capacity to purposefully integrate, build, and reconfigure internal and external competences in response to rapid environmental change. Central to the theory is the idea that organisations must continuously sense changes in the environment, seize emerging opportunities, and reconfigure resources and operational routines to sustain performance over time. ^14^

The theory rests on several key assumptions. It assumes that business environments are dynamic rather than stable, that competitive advantages are temporary rather than permanent, and that managerial cognition and strategic decision-making play a critical role in shaping organisational responses to uncertainty. It also assumes heterogeneity among firms, meaning that organisations differ in their ability to develop and deploy dynamic capabilities due to variations in leadership, learning processes, and organisational routines. ^15, 16^

Dynamic capabilities theory explains strategic thinking and entrepreneurial orientation as higher-order strategic attributes that enable the development and deployment of sensing, seizing, and reconfiguring capabilities. Strategic thinking supports environmental interpretation, long-term orientation, and coherent decision-making, which are essential for sensing complex market and technological shifts. Entrepreneurial orientation reinforces this process by encouraging proactiveness, innovativeness, and calculated risk-taking, which facilitate opportunity seizing and experimentation. Together, these strategic attributes shape how manufacturing SMEs recognise threats and opportunities, mobilise resources, and adapt strategic trajectories across different institutional and market contexts.

The relevance of dynamic capabilities theory to this study lies in its ability to explain how strategic thinking and entrepreneurial orientation move beyond static resource endowments to become drivers of adaptive action. The theory provides a robust conceptual lens for understanding how manufacturing SMEs transform strategic cognition and entrepreneurial behaviour into adaptive resilience through continuous reconfiguration, particularly in environments characterised by technological disruption, supply chain volatility, and regulatory change.

The review is also grounded on Organisational resilience theory. The theory posits that resilience is not limited to the ability of organisations to absorb shocks and recover to a prior state, but also encompasses the capacity to adapt, learn, and transform in response to ongoing and unforeseen disruptions. ^17^

The theory assumes that organisations operate as complex adaptive systems exposed to continuous stressors and episodic shocks. It assumes that uncertainty is inherent rather than exceptional, that disruptions can generate both threats and opportunities, and that resilience emerges from the interaction between organisational structures, processes, and human agency. A further assumption is that resilience is dynamic and path-dependent, shaped by prior learning, strategic choices, and institutional environments. ^18^

Organisational resilience theory explains adaptive resilience as an outcome of proactive and forward-looking organisational behaviour rather than reactive crisis management. Within this framework, resilience reflects the ability of firms to adjust strategies, reconfigure operations, and renew competitive positioning over time. Strategic thinking contributes to resilience by enabling sense-making, anticipation, and strategic coherence under uncertainty, while entrepreneurial orientation strengthens resilience by fostering innovation, flexibility, and opportunity-driven responses to disruption. These mechanisms allow manufacturing SMEs to move beyond short-term recovery towards sustained adaptability across varying institutional and market conditions.

The relevance of organisational resilience theory to this study lies in its emphasis on adaptation and transformation as core elements of resilience. The theory aligns with the study’s focus on adaptive resilience as a long-term strategic outcome shaped by strategic and entrepreneurial capabilities. By anchoring the dependent variable, organisational resilience theory provides a coherent framework for understanding how manufacturing SMEs survive, adjust, and evolve in volatile global manufacturing environments, thereby complementing the process-oriented logic of dynamic capabilities theory.

## Literature review

This section discusses integrated ways through which strategic thinking and entrepreneurial orientation jointly contribute to adaptive resilience in global manufacturing SMEs through dynamic capabilities, with each way articulated through four analytically distinct subthemes. The discussion is framed to capture variation across institutional and market contexts as shown in Table 2.

**Table 2.** Strategic capabilities supporting adaptive resilience in manufacturing SMEs.

Strategic capability	Key findings	Contradictions/Variations	Research gaps	Policy implications
Strategic Environmental Sensing & Opportunity Anticipation	SMEs leverage strategic thinking and entrepreneurial orientation to sense weak signals, interpret complexity, and anticipate change across institutional contexts. ^12, 19, 45^ Evidence from Germany, South Korea, India, and Chile highlights regulatory foresight, proactive scanning, and context-sensitive intelligence gathering. ^26, 27, 34, 35, 39, 46^	Some studies emphasise managerial cognition and interpretive skill, while others highlight network embeddedness, learning routines, and absorptive capacity. ^23, 25, 30, 33^	Limited longitudinal studies linking sensing to sustained resilience; few cross-regional comparisons, especially in climate-sensitive and emerging markets. ^42, 46^	Develop foresight platforms, cluster-based data-sharing systems, and structured research–industry collaboration; provide predictable regulatory pathways for sustainability and digital transitions. ^28, 29, 44, 45^
Opportunity Seizing	SMEs combine strategic prioritisation and entrepreneurial risk-taking to commit resources and act decisively under uncertainty. ^48, 68^ Examples include Japanese automotive component SMEs, Italian specialised machinery firms, and Nordic clean-tech SMEs. ^53, 58, 66^	Contrasts exist regarding whether success is driven more by strategic planning or entrepreneurial risk behaviour, especially under institutional variation.	Limited research on cross-context institutional alignment and the long-term effects of risk-taking on resilience.	Encourage alignment with industrial policy, innovation grants, and green financing; support training in opportunity evaluation and risk management. ^64, 68^
Resource Reconfiguration & Organisational Flexibility	SMEs strategically recombine assets, restructure processes, and adopt flexible organisational arrangements to maintain performance under volatility. ^69, 88^ Cases from France, Vietnam, Canada, and Ghana show context-sensitive reconfiguration and incremental adaptation. ^24, 77, 82, 87^	Differences reflect resource availability and institutional constraints: high-tech contexts prioritise structural redesign, resource-limited SMEs emphasise incremental adaptation.	Scarce longitudinal evidence on durability of reconfiguration practices; limited cross-regional comparative studies.	Support technology adoption, skills development, flexible labour policies, modular production, and collaborative innovation platforms. ^71, 24, 76, 78, 87^
Strategic Learning & Knowledge Integration	Reflective cognition and entrepreneurial experimentation foster organisational learning, knowledge transfer, and cumulative adaptive capability. ^85, 106^ Evidence from the UK, Brazil, Netherlands, and Turkey highlights post-crisis reviews, experiential learning, and cross-functional integration. ^93, 97, 101, 105^	Some studies stress internal reflection, others emphasise network learning and external knowledge integration. ^91, 99^	Limited longitudinal studies on institutionalisation of learning; scarce cross-context comparisons across emerging and developed markets.	Strengthen learning ecosystems via industry–university collaboration, shared innovation labs, digital knowledge repositories, and mobility programmes. ^98, 102^
Strategic Renewal & Long-term Adaptability	SMEs periodically reframe strategic intent, embed continuous innovation, and align short-term adaptation with long-term goals to sustain evolution. ^92, 121^ Cases from Switzerland, Finland, Australia, and Rwanda illustrate service-oriented offerings, innovation pipelines, and policy-aligned renewal. ^111, 115, 118, 121^	Contradictions exist regarding whether renewal is primarily driven by internal leadership or institutional opportunity structures. ^109, 117^	Limited evidence on long-term institutionalisation of renewal routines; few cross-regional and longitudinal studies.	Strengthen SME renewal through industrial transition frameworks, long-term innovation financing, foresight programmes, and sectoral skills initiatives. ^108, 119, 121^

### Strategic environmental sensing and opportunity anticipation

Strategic environmental sensing and opportunity anticipation emerge from the interaction between strategic thinking and entrepreneurial orientation as a critical foundation of adaptive resilience in manufacturing SMEs. ^12, 19^ In volatile manufacturing environments, leaders must make sense of fragmented, ambiguous, and rapidly evolving information while maintaining coherence between immediate operational demands and long-term strategic intent. ^20, 21^ Strategic thinking provides the cognitive architecture for interpretation and integration, while entrepreneurial orientation injects alertness, curiosity, and forward momentum into environmental scanning activities. ^22^ Together, these attributes strengthen the sensing dimension of dynamic capabilities by enabling SMEs to recognise weak signals early, interpret their strategic significance, and anticipate trajectories of change across different institutional and cultural contexts.

Cognitive interpretation of environmental complexity reflects the role of strategic thinking in transforming dispersed environmental signals into actionable strategic insight. ^23^ Manufacturing SMEs often face overlapping technological, regulatory, and competitive pressures that cannot be addressed through linear analysis alone. ^24^ Strategic thinking supports holistic pattern recognition, systems-level reasoning, and scenario-based interpretation, allowing leaders to understand how multiple external forces interact. ^25^ A practical example can be observed in German precision-engineering SMEs operating within highly regulated industrial ecosystems. ^26, 27^ Faced with tightening environmental regulations and rapid advances in Industry 4.0 technologies, many such firms strategically interpret regulatory signals not merely as compliance costs but as indicators of future market expectations. ^28^ This cognitive reframing enables early investment in energy-efficient machinery and digital production systems, positioning these SMEs ahead of competitors when sustainability standards become mandatory. ^29^ The ability to cognitively integrate regulatory foresight, technological trends, and competitive positioning illustrates how strategic thinking strengthens environmental interpretation under complexity.

Proactive market and technology scanning highlights the behavioural contribution of entrepreneurial orientation to sensing capabilities. ^30, 31^ While strategic thinking structures interpretation, entrepreneurial orientation drives continuous outward-looking behaviour, experimentation, and early engagement with emerging trends. ^32^ In manufacturing SMEs, this often manifests as active monitoring of customer needs, supplier innovations, and alternative production technologies. ^33^ An illustrative example can be found among electronics manufacturing SMEs in South Korea, where intense competition and short product life cycles dominate. Firms with strong entrepreneurial orientation actively scan global consumer electronics markets and upstream component innovations, enabling early recognition of shifts towards miniaturisation and smart-device integration. ^34, 35^ By proactively engaging with emerging technologies such as advanced semiconductors and embedded systems, these SMEs anticipate demand changes and adjust product development strategies before market saturation occurs. This proactive scanning behaviour reduces strategic surprise and enhances adaptive readiness in fast-moving technological contexts. ^36^

Context-sensitive intelligence gathering reflects the need for sensing practices to align with institutional and infrastructural realities across different environments. ^37^ Strategic thinking enables leaders to recognise that information sources, signal reliability, and scanning mechanisms vary across regions, while entrepreneurial orientation motivates adaptation rather than reliance on standardised approaches. ^38^ In emerging economies, where formal market data may be limited or unreliable, SMEs often depend on relational networks and informal intelligence channels. ^39^ A practical illustration can be seen in manufacturing SMEs within the Indian textile and apparel sector. These firms frequently rely on close relationships with buyers, intermediaries, and local clusters to gather intelligence on shifting export requirements, labour regulations, and fashion trends. ^40^ By adapting sensing practices to local institutional conditions rather than replicating practices from advanced economies, these SMEs enhance their ability to detect relevant signals despite infrastructural constraints. Context-sensitive intelligence gathering therefore strengthens resilience by aligning sensing mechanisms with environmental realities. ^41^

Anticipatory decision framing captures how strategic thinking and entrepreneurial orientation jointly translate early signals into forward-looking strategic choices. ^42, 43^ Strategic thinking aligns long-term intent with emerging environmental cues, ^44^ while entrepreneurial orientation supports timely action despite uncertainty. ^45^ This anticipatory framing reduces vulnerability by allowing SMEs to prepare for disruptions before they fully materialise. A relevant example can be observed in agribusiness manufacturing SMEs in Latin America facing climate variability and supply chain instability. Some food-processing SMEs in Chile, for instance, have used early climate data and global commodity trends to anticipate disruptions in raw material availability. ^46^ Strategic leaders frame these signals within long-term resilience goals, while entrepreneurial orientation encourages diversification of supplier bases and investment in alternative inputs. This anticipatory alignment between strategic intent and early warning signals enables these firms to absorb shocks with less disruption and maintain continuity in production and market supply.

Taken together, these mechanisms demonstrate that strategic environmental sensing and opportunity anticipation are not passive monitoring activities but dynamic, contextually embedded processes. Strategic thinking provides interpretive depth and coherence, entrepreneurial orientation sustains proactive engagement with uncertainty, and their interaction enables manufacturing SMEs to sense change early and accurately. ^45, 47^ Across diverse cultural and institutional settings, this integrated sensing capability forms a critical foundation for adaptive resilience by transforming environmental uncertainty into strategic foresight and preparedness.

### Opportunity seizing through strategic commitment and entrepreneurial action

Opportunity seizing represents the action-oriented dimension of dynamic capabilities, translating sensed opportunities into tangible strategic commitments. ^48^ In manufacturing SMEs, this process depends on the alignment between strategic thinking, which provides clarity, prioritisation, and long-term coherence, and entrepreneurial orientation, which energises decisive action under uncertainty. ^38, 49^ Together, these attributes enable firms to commit resources, accept calculated risks, respond swiftly to emerging opportunities, and align strategic choices with institutional conditions across diverse global contexts. Through this interaction, opportunity seizing becomes a disciplined yet entrepreneurial process that supports adaptive resilience rather than impulsive decision-making.

Strategic resource prioritisation reflects the role of strategic thinking in guiding how manufacturing SMEs allocate scarce resources toward initiatives that enhance adaptability. ^50^ Given constraints in capital, skills, and technological capacity, SMEs cannot pursue all opportunities simultaneously. ^51^ Strategic thinking enables leaders to evaluate opportunities against long-term strategic intent and resilience objectives, ensuring that resource commitments reinforce future adaptability rather than short-term gains. ^52^ A practical example can be observed in Japanese manufacturing SMEs within the automotive supply chain. Faced with the transition toward electric vehicles, many small component manufacturers strategically prioritised investments in battery-related components and lightweight materials rather than expanding legacy internal combustion technologies. ^53, 54^ Despite limited resources, deliberate prioritisation allowed these firms to align investment decisions with anticipated industry transformation, strengthening their strategic position and resilience in the face of structural change.

Calculated risk-taking behaviour illustrates how entrepreneurial orientation complements strategic thinking by encouraging firms to act despite uncertainty. ^8, 55^ Manufacturing environments often involve irreversible investments, making risk aversion a common barrier to adaptation. ^56^ Entrepreneurial orientation reframes risk as manageable and opportunity-driven rather than purely threatening. ^57^ An illustrative case can be found among manufacturing SMEs in Italy’s industrial districts, particularly in specialised machinery and design-intensive production. These firms often undertake calculated risks by investing in customised production technologies and niche product innovations without guaranteed demand. ^58^ Strategic thinking ensures that such risks are aligned with core competencies and long-term positioning, while entrepreneurial orientation supports commitment to innovation despite uncertain market reception. ^59^ This balanced approach enables SMEs to reposition themselves in high-value niches, reinforcing resilience through differentiation rather than cost competition.

Speed of strategic response highlights the importance of timely execution in opportunity seizing, especially in fast-moving manufacturing sectors. ^60^ Strategic thinking provides a forward-looking roadmap, while entrepreneurial orientation drives swift mobilisation of resources and rapid decision-making. ^61^ Together, they reduce delays that could erode first-mover advantages. A relevant example can be drawn from manufacturing SMEs in China’s consumer goods sector, where market preferences and competitive dynamics shift rapidly. ^62^ Firms with strong entrepreneurial orientation and strategic clarity have demonstrated the ability to rapidly retool production lines and adjust product specifications in response to emerging consumer trends. ^63^ By combining anticipatory planning with rapid execution, these SMEs exploit short-lived market windows, sustaining competitiveness in highly dynamic environments.

Institutionally aligned strategic choices reflect how opportunity seizing must adapt to variations in policy frameworks, financial systems, and market maturity across regions. ^64^ Strategic thinking enables leaders to understand institutional incentives and constraints, while entrepreneurial orientation motivates proactive engagement with these structures. ^65^ In Nordic countries, such as Sweden, manufacturing SMEs often align opportunity seizing with strong policy support for sustainability and innovation. ^66^ Firms actively leverage government grants, green financing instruments, and collaborative innovation platforms to pursue advanced manufacturing and clean technology initiatives. ^67^ Strategic thinking ensures alignment with national industrial strategies, while entrepreneurial orientation drives active participation in policy-enabled opportunities. ^68^ This institutional alignment enhances resilience by embedding opportunity seizing within supportive regulatory and financial ecosystems.

Collectively, these dimensions demonstrate that opportunity seizing in manufacturing SMEs is neither purely analytical nor purely opportunistic. Strategic thinking provides discipline, coherence, and long-term alignment, while entrepreneurial orientation injects action, speed, and risk tolerance. Across diverse cultural and institutional contexts, their interaction enables SMEs to commit resources, embrace uncertainty, and act decisively, transforming environmental opportunities into adaptive resilience and sustained strategic renewal.

### Resource reconfiguration and organisational flexibility

Resource reconfiguration and organisational flexibility constitute the transformative core of adaptive resilience in manufacturing SMEs. ^69^ While environmental sensing and opportunity seizing determine *what* to respond to and *when* to act, reconfiguration determines *how* firms realign internal and external resources to sustain performance under changing conditions. ^70^ This process relies on the interaction between strategic cognition, which provides deliberate direction and coherence, and entrepreneurial behaviour, which enables experimentation, flexibility, and action under uncertainty. Together, these attributes allow manufacturing SMEs to continuously recombine assets, reshape organisational arrangements, and adapt practices to diverse institutional contexts. ^71^

Strategic recombination of assets reflects the role of strategic thinking in deliberately restructuring production systems, supplier relationships, and technology portfolios to align with evolving competitive conditions. ^72, 73^ Rather than acquiring entirely new resources, resilient SMEs often reconfigure existing assets in novel ways. A practical example can be observed among manufacturing SMEs in France’s advanced materials sector. Facing increased cost pressures and demand for customised solutions, several firms strategically restructured supplier networks by shifting from transactional sourcing to collaborative partnerships with specialised material providers. At the same time, production processes were reorganised to support small-batch, high-precision manufacturing. Strategic thinking guided these recombination decisions by aligning operational changes with long-term positioning in high-value markets, enabling adaptation without excessive capital expenditure. ^24^

Innovative experimentation highlights the contribution of entrepreneurial orientation to reconfiguration processes. ^45, 74^ Entrepreneurial behaviour encourages trial-and-error learning, piloting of new processes, and incremental innovation within manufacturing operations. ^75^ This experimentation reduces rigidity and enables firms to adapt gradually rather than through disruptive overhauls. An illustrative example can be found among manufacturing SMEs in Vietnam’s furniture and wood-processing industry. As global buyers increasingly demanded sustainable and customised products, entrepreneurial-oriented firms experimented with alternative materials, modular designs, and digital fabrication techniques. ^76, 77^ These initiatives were often implemented on a small scale to test feasibility before broader rollout. Through continuous experimentation, these SMEs incrementally adapted production practices, balancing innovation with operational stability in a resource-constrained environment. ^78^

Flexible organisational structures demonstrate how strategic cognition and entrepreneurial behaviour translate into internal adaptability. ^79^ Strategic thinking supports the redesign of organisational routines and coordination mechanisms, while entrepreneurial orientation fosters openness to role flexibility and cross-functional collaboration. ^80^ In manufacturing SMEs, rigid hierarchies can slow response to volatility, making structural flexibility a key resilience mechanism. ^81^ A relevant example can be seen in manufacturing SMEs in Canada’s advanced manufacturing clusters. Many firms have adopted flatter organisational structures and project-based teams that allow rapid reallocation of responsibilities in response to shifts in customer demand or supply chain disruptions. ^82, 83^ Strategic leadership ensures alignment with overarching goals, while entrepreneurial culture empowers employees to take initiative, enabling swift operational adjustment without undermining strategic coherence. ^84^

Contextual adaptation of reconfiguration practices underscores the influence of institutional and structural conditions on how SMEs reconfigure resources. ^85^ Strategic thinking enables leaders to understand regulatory constraints and labour market dynamics, while entrepreneurial orientation encourages creative navigation of these conditions. ^86^ In sub-Saharan Africa, for instance, manufacturing SMEs often face limited access to advanced technology and rigid labour regulations. ^87^ A practical illustration can be found among manufacturing SMEs in Ghana’s agro-processing sector, where firms adapt reconfiguration practices by leveraging flexible labour arrangements, local sourcing, and incremental mechanisation. Rather than adopting capital-intensive technologies common in advanced economies, these SMEs reconfigure operations in ways that align with institutional realities, enhancing resilience through contextual fit rather than replication of foreign models. ^87, 88^

Taken together, resource reconfiguration and organisational flexibility illustrate how adaptive resilience emerges from continuous internal transformation rather than static efficiency. Strategic cognition provides purposeful direction for recombination, entrepreneurial behaviour sustains experimentation and flexibility, and contextual awareness ensures institutional alignment. Across diverse manufacturing environments, this integrated reconfiguration capability enables SMEs to respond to volatility, sustain competitiveness, and evolve strategically over time.

### Strategic learning and knowledge integration

Strategic learning and knowledge integration form the cumulative mechanism through which manufacturing SMEs convert experience into sustained adaptive capacity. ^85^ In volatile environments, resilience depends not only on timely sensing, seizing, and reconfiguration, but also on the ability to learn systematically from both success and failure. ^89^ Strategic thinking provides the reflective discipline required to interpret past actions and outcomes, while entrepreneurial orientation energises experiential learning through experimentation and innovation. ^90, 91^ Together, these attributes strengthen the learning dimension of dynamic capabilities by embedding continuous knowledge creation, integration, and transfer within organisational practice across diverse contexts.

Reflective strategic learning captures the role of strategic thinking in enabling deliberate evaluation of prior disruptions, strategic choices, and performance consequences. ^91^ Rather than treating shocks as isolated events, resilient manufacturing SMEs engage in structured reflection to extract strategic lessons that inform future decisions. ^92^ A practical example can be observed among manufacturing SMEs in the United Kingdom’s aerospace and precision engineering sector. Following supply chain disruptions linked to geopolitical uncertainty and pandemic-related shocks, several firms systematically reviewed procurement strategies, inventory policies, and supplier dependencies. Strategic leaders facilitated post-disruption reviews that linked operational outcomes to strategic assumptions, enabling recalibration of sourcing strategies and investment priorities. This reflective process strengthened preparedness for future disruptions by institutionalising learning rather than relying on ad hoc responses. ^93, 94^

Learning through entrepreneurial experimentation highlights the behavioural contribution of entrepreneurial orientation to organisational learning. ^74^ Experimentation allows SMEs to generate new knowledge under conditions of uncertainty where prior experience may be insufficient. ^95^ Entrepreneurial orientation legitimises trial-and-error initiatives, pilot projects, and exploratory innovation, even when outcomes are uncertain. ^96^ An illustrative example can be found in manufacturing SMEs within Brazil’s metalworking and machinery sector. Firms pursuing product diversification often engage in small-scale experimentation with new production techniques and customised solutions for niche markets. ^97^ These experiments generate practical knowledge about cost structures, customer preferences, and technological feasibility, which informs subsequent scaling decisions. Through repeated cycles of experimentation, SMEs build a repository of experiential knowledge that enhances adaptive decision-making.

Knowledge integration across functions and networks reflects the need to align learning generated in different organisational domains. ^98^ Strategic thinking supports the synthesis of insights from production, supply chains, and markets, while entrepreneurial orientation encourages boundary-spanning collaboration and information sharing. ^99^ In manufacturing SMEs, silos can undermine resilience by fragmenting knowledge. ^100^ A relevant example can be seen among manufacturing SMEs in the Netherlands’ high-tech manufacturing clusters. These firms actively integrate knowledge across engineering, operations, and customer-facing functions through cross-functional teams and collaborative platforms. ^101^ External networks with suppliers, research institutions, and industry associations further enrich learning processes. By integrating internal and external knowledge, these SMEs ensure that strategic insights translate into coordinated operational and market responses. ^102^

Cross-context knowledge transfer underscores how learning mechanisms must adapt to institutional diversity and resource variation across regions. ^103^ Strategic thinking enables leaders to recognise contextual differences, while entrepreneurial orientation supports flexible adaptation of learned practices rather than rigid replication. ^104^ A practical illustration can be found in multinational manufacturing SMEs originating from Turkey that operate across European and Middle Eastern markets. These firms transfer production know-how and process innovations across units while adapting learning routines to local regulatory frameworks, labour skills, and market volatility. ^105^ Rather than standardising practices wholesale, strategic leaders encourage contextual modification, ensuring that transferred knowledge remains relevant and effective. This adaptive transfer enhances resilience by leveraging accumulated learning while respecting institutional constraints. ^106^

Collectively, strategic learning and knowledge integration demonstrate that adaptive resilience in manufacturing SMEs is reinforced through continuous, multi-level learning processes. Strategic thinking provides structure and coherence to reflection, entrepreneurial orientation sustains experiential knowledge creation, and integration mechanisms ensure alignment across functions, networks, and contexts. Across diverse manufacturing environments, this learning capability enables SMEs to refine strategies, improve responsiveness, and sustain long-term adaptability under persistent uncertainty.

### Strategic renewal and long-term adaptability

Strategic renewal and long-term adaptability represent the culmination of adaptive resilience in manufacturing SMEs, extending resilience beyond short-term recovery towards sustained strategic evolution. ^92^ In environments characterised by persistent uncertainty and structural change, resilience depends on the capacity to periodically reassess strategic direction, renew value propositions, and embed adaptability within organisational routines. ^107^ Strategic thinking provides the cognitive foundation for reassessing purpose and positioning, while entrepreneurial orientation sustains momentum through continuous innovation and opportunity-driven behaviour. ^108^ Together, these attributes enable manufacturing SMEs to pursue renewal as an ongoing strategic process rather than a reactive adjustment to disruption.

Reframing of strategic intent reflects the role of strategic thinking in enabling firms to periodically revisit and redefine their competitive positioning and value propositions. ^109^ As markets evolve, strategic intent must shift to remain relevant. ^110^ A practical example can be observed among manufacturing SMEs in Switzerland’s precision instruments sector. Facing increasing global competition and commoditisation pressures, several firms have strategically reframed their intent from product-based competition towards solution-oriented offerings that integrate services, customisation, and long-term client partnerships. ^111^ Strategic thinking enables leaders to reassess where and how value is created, ensuring that renewal efforts align with emerging market expectations rather than past success formulas. ^112^

Sustained innovativeness highlights how entrepreneurial orientation embeds innovation as a continuous organisational practice. ^113^ Rather than treating innovation as a response to crisis, resilient SMEs institutionalise experimentation, creativity, and opportunity recognition within everyday operations. ^114^ An illustrative example can be found among manufacturing SMEs in Finland’s clean technology and advanced manufacturing sectors. ^115^ These firms maintain ongoing innovation pipelines that include incremental process improvements, product upgrades, and exploration of new materials. Entrepreneurial orientation legitimises continuous investment in innovation, even during periods of relative stability, ensuring that firms remain prepared for future disruption and capable of strategic renewal.

Alignment of short-term adaptation with long-term vision underscores the importance of coherence between immediate responses to volatility and overarching strategic objectives. ^116^ Strategic thinking ensures that adaptive actions taken under pressure reinforce, rather than undermine, long-term goals, while entrepreneurial orientation enables rapid execution without strategic drift. ^117^ A relevant example can be drawn from manufacturing SMEs in Australia’s food and beverage processing industry. During periods of supply chain disruption, firms have implemented short-term operational adjustments such as sourcing alternative inputs or modifying production schedules. ^118^ Strategic leaders frame these adaptations within a long-term vision of market diversification and brand positioning, ensuring that immediate responses contribute to strategic progression rather than opportunistic fragmentation.

Institutional embeddedness of renewal processes highlights how national policies, cultural norms, and market structures shape the pace and form of strategic transformation. ^119^ Strategic thinking enables SMEs to interpret institutional signals, while entrepreneurial orientation drives proactive engagement with supportive or constraining environments. ^120^ An illustrative case can be seen among manufacturing SMEs in Rwanda’s light manufacturing sector, where government-led industrialisation policies, special economic zones, and skills development initiatives shape strategic renewal pathways. Firms align renewal efforts with national development priorities, leveraging policy incentives while adapting business models to local market conditions. ^121^ This institutional embeddedness enhances the sustainability of renewal by grounding strategic transformation within supportive socio-economic structures.

Taken together, strategic renewal and long-term adaptability demonstrate how resilience in manufacturing SMEs evolves into a forward-looking strategic capability. Strategic thinking enables periodic reframing of intent and coherence across time horizons, entrepreneurial orientation sustains continuous innovativeness, and institutional alignment ensures contextual viability. Across diverse global contexts, this integrated renewal process allows manufacturing SMEs to evolve strategically, maintain relevance, and sustain adaptive resilience in the face of enduring uncertainty.

## Discussion of literature review

This section synthesises the literature on strategic capabilities in manufacturing SMEs by comparing prior findings, identifying contradictions and research gaps, and outlining policy directions that support long-term adaptive resilience. This is shown in Table 3.

**Table 3.** Critical analysis of strategic capabilities and adaptive resilience in manufacturing SMEs.

Strategic capability	Key findings	Contradictions/Variations	Research gaps	Policy implications
Strategic Environmental Sensing & Opportunity Anticipation	SMEs combining strategic cognition and entrepreneurial proactiveness identify early regulatory, technological, and market signals to gain competitive advantage. ^20, 25, 45, 47^ Evidence from Germany, South Korea, and India confirms cognitive reframing, proactive scanning, and network-based intelligence. ^26, 29, 34– 41^	Some studies prioritise managerial cognition and interpretive skill, others emphasise structural embeddedness and absorptive capacity. ^23– 25, 30– 33^	Limited longitudinal evidence linking sensing to sustained resilience; few cross-regional comparisons, especially in climate-sensitive sectors. ^42– 46^	Support foresight platforms, cluster-based data sharing, research–industry collaboration; provide predictable regulatory pathways to encourage early investment. ^28, 29, 44, 45^
Resource Reconfiguration & Organisational Flexibility	Strategic cognition and entrepreneurial behaviour enable asset realignment, process adaptation, and structural flexibility under volatility. ^69, 70, 71^ Case studies in France, Vietnam, and Canada highlight strategic recombination, incremental experimentation, and flat organisational structures. ^24, 72, 73, 76– 78, 82– 84^	Emphasis differs by context: resource-rich/high-tech firms prioritise structural redesign; resource-constrained SMEs focus on incremental adaptation and flexible labour. ^85– 88^	Limited longitudinal studies on sustainability of reconfiguration practices; few comparative studies in emerging/sub-Saharan African contexts. ^85– 88^	Provide technology adoption support, skills development, flexible labour policies, collaborative innovation platforms, and modular production incentives. ^24, 71, 76, 78, 87^
Strategic Learning & Knowledge Integration	Reflective cognition and entrepreneurial experimentation reinforce resilience; post-crisis reflection, experimentation, and cross-functional integration improve preparedness and coordination. ^85, 102^ Evidence from the UK, Brazil, Netherlands, and Turkey supports internal learning and network-based knowledge transfer.	Some studies emphasise internal reflection, others highlight inter-firm learning and network embeddedness. ^91, 99^	Lack of longitudinal evidence on institutionalising lessons; limited cross-context comparative research. ^103, 106^	Strengthen SME learning ecosystems via industry–university collaboration, shared innovation labs, digital knowledge repositories, and mobility programmes. ^98, 102^
Strategic Renewal & Long-term Adaptability	Periodic reassessment, continuous innovation, and entrepreneurial dynamism support sustained SME competitiveness. ^92, 107– 118^ Evidence from Switzerland, Finland, and Australia demonstrates reframed strategic intent, innovation pipelines, and alignment of short-term adaptation with long-term positioning.	Debate over whether renewal is driven more by internal strategic leadership or institutional opportunity structures. ^109– 117^	Limited longitudinal and cross-regional studies on institutionalising renewal; few analyses of long-term cumulative competitiveness vs. overstretch. ^92, 107, 119– 121^	Promote predictable industrial transition frameworks, long-term innovation financing, sectoral foresight programmes, and public–private experimentation platforms. ^108, 119– 121^

### Strategic environmental sensing, opportunity anticipation and adaptive resilience in manufacturing SMEs

The literature largely supports the review’s argument that strategic environmental sensing emerges from the joint influence of strategic cognition and entrepreneurial proactiveness, with multiple studies confirming that SMEs capable of interpreting regulatory, technological, and market signals early tend to secure competitive positioning advantages. ^20, 25, 45, 47^ Empirical work on industrial SMEs in Germany similarly shows that regulatory foresight linked to sustainability transitions often stimulates innovation rather than compliance-only responses, reinforcing the review’s interpretation of cognitive reframing. ^26, 29^ Studies examining export-driven technology sectors in South Korea also align with findings that entrepreneurial orientation promotes continuous scanning and rapid product adaptation in environments characterised by short innovation cycles. ^34– 36^ Research on relational market systems in India corroborates the observation that SMEs substitute formal market intelligence with network-based information flows, supporting institutional contingency arguments in sensing theory. ^37– 41^ Yet some contradictions persist in prior scholarship. Certain studies attribute opportunity recognition primarily to managerial cognition and interpretive skill, while others emphasise structural embeddedness, learning routines, and absorptive capacity as stronger predictors of sensing effectiveness. Divergent findings often reflect sectoral differences, technological intensity, and institutional predictability; regulated engineering industries tend to reward interpretive foresight, whereas volatile consumer-technology sectors favour behavioural experimentation and rapid iteration. ^23– 25, 30– 33^

Important research gaps remain, particularly the shortage of longitudinal evidence linking sensing practices to sustained resilience rather than short-term adaptation. ^42– 45^ Cross-regional comparisons are still limited, even though emerging evidence from climate-sensitive manufacturing systems such as agri-processing SMEs in Chile indicates that environmental sensing interacts strongly with supply volatility and ecological risk. ^46^ Future research should therefore integrate multi-level designs connecting managerial cognition, organisational routines, and ecosystem information infrastructures, while mixed-method approaches could better capture informal sensing mechanisms often invisible in survey-based research. ^37– 41^ Policy interventions should focus on strengthening institutional support for anticipatory intelligence in SMEs, including publicly funded foresight platforms, cluster-based data-sharing systems, and structured collaboration between research institutions and manufacturing networks. Governments can also improve sensing effectiveness by issuing predictable regulatory transition pathways in sustainability and digital transformation, thereby reducing uncertainty and encouraging early strategic investment. ^28, 29, 44, 45^ Such coordinated policy frameworks would help SMEs convert fragmented environmental signals into actionable foresight, reinforcing adaptive resilience across diverse manufacturing environments.

### Opportunity seizing and adaptive Resilience in manufacturing SMEs

The literature largely supports the review’s argument that strategic environmental sensing emerges from the joint influence of strategic cognition and entrepreneurial proactiveness, with multiple studies confirming that SMEs capable of interpreting regulatory, technological, and market signals early tend to secure competitive positioning advantages. ^20, 25, 45, 47^ Empirical work on industrial SMEs in Germany similarly shows that regulatory foresight linked to sustainability transitions often stimulates innovation rather than compliance-only responses, reinforcing the review’s interpretation of cognitive reframing. ^26, 29^ Studies examining export-driven technology sectors in South Korea also align with findings that entrepreneurial orientation promotes continuous scanning and rapid product adaptation in environments characterised by short innovation cycles. ^34– 36^ Research on relational market systems in India corroborates the observation that SMEs substitute formal market intelligence with network-based information flows, supporting institutional contingency arguments in sensing theory. ^37– 41^ Yet some contradictions persist in prior scholarship. Certain studies attribute opportunity recognition primarily to managerial cognition and interpretive skill, while others emphasise structural embeddedness, learning routines, and absorptive capacity as stronger predictors of sensing effectiveness. Divergent findings often reflect sectoral differences, technological intensity, and institutional predictability; regulated engineering industries tend to reward interpretive foresight, whereas volatile consumer-technology sectors favour behavioural experimentation and rapid iteration. ^23– 25, 30– 33^

Important research gaps remain, particularly the shortage of longitudinal evidence linking sensing practices to sustained resilience rather than short-term adaptation. ^42– 45^ Cross-regional comparisons are still limited, even though emerging evidence from climate-sensitive manufacturing systems such as agri-processing SMEs in Chile indicates that environmental sensing interacts strongly with supply volatility and ecological risk. ^46^ Future research should therefore integrate multi-level designs connecting managerial cognition, organisational routines, and ecosystem information infrastructures, while mixed-method approaches could better capture informal sensing mechanisms often invisible in survey-based research. ^37– 41^ Policy interventions should focus on strengthening institutional support for anticipatory intelligence in SMEs, including publicly funded foresight platforms, cluster-based data-sharing systems, and structured collaboration between research institutions and manufacturing networks. Governments can also improve sensing effectiveness by issuing predictable regulatory transition pathways in sustainability and digital transformation, thereby reducing uncertainty and encouraging early strategic investment. ^28, 29, 44, 45^ Such coordinated policy frameworks would help SMEs convert fragmented environmental signals into actionable foresight, reinforcing adaptive resilience across diverse manufacturing environments.

### Resource reconfiguration and organisational flexibility in manufacturing SMEs

The literature consistently supports the view that resource reconfiguration and organisational flexibility are central to adaptive resilience, with multiple studies highlighting that SMEs combining strategic cognition with entrepreneurial behaviour can realign assets, restructure operations, and adjust organisational arrangements to sustain performance under environmental volatility. ^69, 70, 71^ Comparative evidence across diverse contexts shows convergence in findings: European SMEs in France strategically recombine supplier networks and production processes to support customised, high-value manufacturing, ^24, 72, 73^ while Asian SMEs in Vietnam leverage incremental experimentation to adapt materials and modular production techniques. ^76– 78^ Research in North America, particularly Canada, similarly illustrates that flatter organisational structures and project-based teams enhance internal adaptability and responsiveness. ^82– 84^ These studies collectively indicate that deliberate strategic planning provides coherence for internal transformation, while entrepreneurial experimentation drives flexibility, enabling SMEs to balance innovation with operational stability. Differences in emphasis across studies often reflect institutional and sectoral conditions, with resource-rich, technology-intensive contexts prioritising structural redesign and asset recombination, whereas resource-constrained environments highlight incremental adaptation, local sourcing, and flexible labour arrangements as mechanisms of resilience. ^85– 88^

Despite widespread agreement on the importance of reconfiguration and flexibility, the literature reveals gaps and areas requiring further investigation. Longitudinal evidence on the sustainability of reconfiguration practices remains limited, particularly in SMEs operating under chronic resource constraints or regulatory uncertainty, leaving questions about the durability of adaptive routines. Cross-regional comparative studies are scarce, especially in emerging and sub-Saharan African contexts, where institutional constraints, labour dynamics, and technology access significantly shape reconfiguration strategies. ^85– 88^ Future research should therefore adopt multi-level and longitudinal designs to examine how strategic cognition, entrepreneurial experimentation, and contextual adaptation interact over time to sustain competitive advantage. Policy interventions should focus on enabling SMEs to implement adaptive resource practices by providing support for technology adoption, skills development, flexible labour policies, and collaborative platforms for shared innovation. Governments can also incentivise incremental experimentation and modular production approaches through grants, training programmes, and sectoral infrastructure development, ensuring that SMEs can reconfigure resources effectively while aligning with institutional and market realities. ^24, 71, 76, 78, 87^

### Strategic learning, knowledge integration and adaptive resilience in manufacturing SMEs

The literature strongly supports the review’s central claim that organisational learning reinforces adaptive resilience when reflective cognition is combined with entrepreneurial experimentation, with prior studies similarly linking post-disruption reflection to improved strategic preparedness and supply-chain robustness. ^85, 89– 94^ Empirical research on industrial SMEs in United Kingdom confirms that structured post-crisis reviews often lead to revised sourcing strategies, risk diversification, and improved strategic foresight, reinforcing the importance of reflective learning routines. Studies of innovation-driven SMEs in Brazil likewise align with the argument that entrepreneurial experimentation accelerates knowledge creation and strategic renewal in uncertain markets. ^74, 95– 97^ Evidence from collaborative innovation systems such as those observed in Netherlands also supports the view that cross-functional and network-based knowledge integration enhances responsiveness and coordination in technology-intensive manufacturing contexts. ^98– 102^ Nonetheless, contradictions remain concerning whether learning outcomes depend more on internal reflection or external knowledge exchange. Some studies find that structured internal review processes drive strategic adaptation, whereas others show that network embeddedness and inter-firm learning exert stronger influence on innovation outcomes. Divergent findings often reflect industry knowledge intensity and cluster maturity; high-tech ecosystems reward network integration, while resource-constrained SMEs rely more heavily on internal experiential learning. ^74, 91– 94, 95– 99^

Significant research gaps persist in understanding how learning mechanisms evolve longitudinally and across institutional environments. Evidence remains limited on how SMEs institutionalise lessons over time rather than reverting to reactive routines after immediate crises subside. ^103– 106^ Cross-context comparative work is also scarce, even though emerging insights from internationally active manufacturing SMEs in Turkey indicate that adaptive transfer of knowledge across regulatory and market environments strongly influences resilience trajectories. Future research should therefore adopt longitudinal and multi-level approaches linking managerial cognition, experimentation routines, and network learning infrastructures, while also examining how digital knowledge platforms reshape SME learning dynamics. Policy interventions should prioritise strengthening SME learning ecosystems through subsidised industry–university collaboration, shared innovation laboratories, and structured post-crisis review frameworks supported by public agencies. Governments can also promote knowledge integration by funding cluster-based digital knowledge repositories, mobility programmes for technical skills exchange, and advisory services that help SMEs convert experiential insights into formalised strategic routines. ^98– 102^ Such policy alignment would enable manufacturing SMEs to transform episodic experience into cumulative strategic capability, reinforcing long-term adaptive resilience across diverse institutional environments.

### Strategic renewal, long-term adaptability and adaptive resilience in manufacturing SMEs

Prior research largely supports the review’s argument that strategic renewal emerges from the interaction of reflective strategic cognition and sustained entrepreneurial dynamism, with studies consistently linking periodic reassessment of competitive positioning to long-term SME survival in structurally shifting industries. ^92, 107– 112^ Evidence from high-precision manufacturing systems in Switzerland similarly shows that SMEs shifting from product competition to service-embedded solutions strengthen differentiation and customer lock-in, reinforcing the importance of reframing strategic intent under commoditisation pressures. Research on innovation-intensive sectors in Finland aligns with the review’s claim that continuous innovation pipelines foster renewal readiness even during stable periods, confirming that entrepreneurial orientation functions as a stabilising force for long-term adaptability rather than merely a crisis response. ^113– 115^ Empirical studies of export-exposed food processing firms in Australia also support the emphasis on aligning short-term adjustments with long-term positioning, demonstrating that adaptive responses strengthen competitiveness only when embedded within coherent strategic trajectories. ^116– 118^ Despite this convergence, contradictions remain in the literature regarding whether renewal is driven primarily by internal strategic leadership or by institutional opportunity structures. Some studies attribute renewal success to visionary managerial cognition, whereas others emphasise industrial policy alignment, access to innovation systems, and ecosystem support as stronger predictors of sustained transformation. ^109– 117^

Important research gaps remain, particularly concerning how SMEs institutionalise renewal routines over time rather than relying on episodic transformation triggered by crisis. ^92, 107, 119^ Cross-regional comparisons are still limited, even though emerging evidence from policy-guided industrial systems such as those in Rwanda suggests that national development strategies, skills initiatives, and industrial zones significantly shape SME renewal pathways. ^120, 121^ Future research should therefore integrate dynamic capability theory with institutional development perspectives to explain how policy environments influence the durability and direction of strategic renewal. Longitudinal and ecosystem-level analyses would also clarify whether continuous innovativeness leads to cumulative competitiveness or strategic overstretch in resource-constrained firms. Policy interventions should prioritise strengthening SME renewal capacity through predictable industrial transition frameworks, long-term innovation financing instruments, and structured public–private platforms that support experimentation beyond immediate productivity gains. Governments can also enhance renewal by embedding SMEs in sectoral foresight programmes, export upgrading initiatives, and skills transformation strategies aligned with technological shifts. ^108, 119– 121^ Such coordinated policy ecosystems would enable manufacturing SMEs to convert episodic adaptation into sustained strategic evolution, reinforcing long-term adaptive resilience across diverse institutional environments.

### Theoretical implications

The review examines relationship between literature review and the underpinning theoretical framework thereby identifying the persistent challenges and eminent gaps as shown in Table 4.

**Table 4.** Key mechanisms of adaptive resilience in manufacturing SMEs.

Theme	Strategic thinking role	Entrepreneurial orientation role	Illustrative example	Related theory	Policy implications
Strategic environmental sensing & opportunity anticipation	Integrates and interprets fragmented environmental signals; supports scenario-based analysis	Drives proactive scanning, curiosity, and early engagement with emerging trends	German precision-engineering SMEs anticipating regulatory and Industry 4.0 shifts	Dynamic capabilities & Organisational resilience theory	Support market intelligence platforms, data access initiatives, SME training in foresight skills
Opportunity seizing through strategic commitment	Guides prioritisation, resource allocation, and long-term alignment	Encourages calculated risk-taking, speed, and decisive action under uncertainty	Japanese automotive SMEs investing in EV components	Dynamic capabilities theory	Provide innovation grants, risk-sharing schemes, incentives for first-mover initiatives
Resource reconfiguration & organisational flexibility	Directs asset recombination, process redesign, and structural alignment	Enables experimentation, incremental innovation, and flexible internal arrangements	French advanced materials SMEs restructuring supplier networks; Vietnamese furniture SMEs piloting modular designs	Dynamic capabilities & Organisational resilience theory	Facilitate access to flexible financing, encourage adaptive workforce policies, SME advisory support
Strategic learning & knowledge integration	Structures reflective learning, synthesises internal insights	Promotes experiential learning, cross-functional and network knowledge sharing	UK aerospace SMEs reviewing supply chains post-disruption; Turkish SMEs transferring knowledge across regions	Dynamic capabilities & Organisational resilience theory	Promote knowledge-sharing networks, cross-border collaboration, R&D and training incentives
Strategic renewal & long-term adaptability	Reframes strategic intent, ensures coherence between short-term actions and long-term goals	Sustains continuous innovation and proactive market adaptation	Swiss precision instruments SMEs shifting to solution-based offerings; Finnish clean-tech SMEs maintaining innovation pipelines	Dynamic capabilities & Organisational resilience theory	Support policy frameworks for industrial renewal, provide innovation ecosystem incentives, long-term SME development programs

### Environmental sensing and opportunity anticipation in manufacturing SMEs: Theoretical alignment

The literature review strongly aligns with the principles of Dynamic Capabilities Theory, illustrating that strategic thinking and entrepreneurial orientation function as higher-order capabilities enabling manufacturing SMEs to sense environmental complexity, seize opportunities, and reconfigure resources under volatile conditions. ^12, 19, 22, 30^ Empirical examples from Germany, South Korea, and India demonstrate that SMEs leverage cognitive interpretation and proactive scanning to anticipate regulatory, technological, and market shifts, consistent with Teece, Pisano, and Shuen’s assertion that competitiveness depends on purposive integration and reconfiguration of internal and external competences. ^14– 16^ Furthermore, strategic learning, knowledge integration, and renewal practices observed in the UK, Brazil, and Finland reflect the dynamic capabilities logic, whereby continuous experimentation, cross-functional knowledge transfer, and iterative innovation enable firms to adapt long-term strategies in response to environmental turbulence. ^85, 97, 115^ Organisational Resilience Theory complements this perspective by emphasizing that adaptive resilience emerges not merely from reactive recovery but from the proactive adjustment of structures, processes, and strategic intent. ^17, 18^ The literature demonstrates that SMEs combining strategic cognition and entrepreneurial behaviour are capable of sustained adaptation, learning from past disruptions, and embedding flexibility within organisational routines, thereby operationalizing resilience as an ongoing strategic outcome rather than episodic crisis response.

Despite this theoretical coherence, persistent challenges and research gaps remain. Contradictions exist regarding the relative influence of internal managerial cognition versus external institutional structures on sensing, opportunity seizing, and renewal outcomes, particularly in resource-constrained or emerging-market contexts. ^23, 37, 109^ Longitudinal evidence on the institutionalization of adaptive routines, cross-contextual transfer of learning, and sustainability of reconfiguration strategies remains limited, creating uncertainty about how SMEs maintain resilience over extended periods. ^42, 103, 119^ Policy interventions are therefore critical to bridging these gaps. Governments can strengthen SME adaptive capacity through structured foresight platforms, innovation grants, and cluster-based knowledge networks that facilitate early sensing and opportunity alignment. ^28, 29, 44^ Supporting technology adoption, skills development, flexible labour arrangements, and sectoral infrastructure further enhances reconfiguration and experimentation capabilities. ^71, 76, 87^ Additionally, embedding SMEs in long-term industrial transition frameworks, sectoral skills initiatives, and export upgrading programmes ensures that adaptive and renewal processes are institutionally supported, transforming episodic adaptation into sustained strategic resilience across diverse manufacturing environments. ^108, 119, 121^

### Opportunity seizing in manufacturing SMEs: Theoretical alignment

The literature on opportunity seizing in manufacturing SMEs strongly aligns with the principles of Dynamic Capabilities Theory, which emphasises the capacity of firms to sense, seize, and transform resources in response to environmental change. ^38, 48, 49^ Strategic thinking and entrepreneurial orientation operate as higher-order capabilities that enable SMEs to prioritise resources, take calculated risks, and execute rapid strategic actions under uncertainty. ^50, 55, 60^ Empirical evidence from Japan, Italy, and China illustrates that deliberate resource allocation, risk-enabled innovation, and swift operational adjustment translate environmental signals into concrete strategic commitments, reflecting the theory’s assertion that competitiveness relies on purposeful integration and deployment of dynamic capabilities. ^14, 15^ Organisational Resilience Theory complements this framework by highlighting that adaptive resilience emerges from proactive, forward-looking behaviours rather than mere reactive responses. ^17, 18^ Strategic thinking provides coherence and alignment with long-term objectives, while entrepreneurial orientation energises opportunity-driven experimentation and institutional engagement, collectively reinforcing SMEs’ ability to convert short-term opportunities into sustained adaptive and strategic outcomes. ^53, 63, 68^

Despite theoretical coherence, persistent gaps remain in understanding the contextual contingencies influencing opportunity seizing. Contradictions in the literature highlight differences in whether opportunity exploitation is primarily shaped by internal managerial cognition or by institutional conditions such as regulatory frameworks, financial systems, and policy incentives. ^64, 65^ Longitudinal evidence on how SMEs institutionalise opportunity-seizing routines for sustained resilience remains scarce, particularly in emerging and resource-constrained markets. ^52, 59^ Policy interventions are therefore crucial to strengthen SME adaptive capacity. Governments can support structured foresight and innovation platforms, provide targeted grants and financial instruments, and promote cluster-based knowledge networks to enhance strategic prioritisation, risk-enabled innovation, and rapid response capabilities. ^66, 67^ Additionally, embedding SMEs within sector-specific transition frameworks and policy-aligned innovation initiatives ensures that opportunity-seizing practices are systematically reinforced, enabling firms to translate environmental opportunities into long-term adaptive resilience and strategic renewal. ^68^

### Resource reconfiguration and organisational flexibility in manufacturing SMEs: Theoretical alignment

The literature on resource reconfiguration and organisational flexibility underscores the centrality of dynamic capabilities theory in explaining how manufacturing SMEs transform internal and external resources to sustain performance under volatile conditions. ^69, 70^ Strategic thinking provides deliberate direction for recombining assets, redesigning production systems, and aligning operational changes with long-term competitive positioning. ^24, 72^ Entrepreneurial orientation complements this by fostering experimentation, incremental innovation, and flexible organisational structures, allowing SMEs to respond rapidly to market and technological shifts. ^45, 80^ Together, these attributes operationalise the dynamic capabilities cycle of sensing, seizing, and reconfiguring, demonstrating how higher-order strategic cognition and proactive behaviour enable SMEs to adapt continuously across diverse institutional and market contexts. ^71, 85^ Organisational resilience theory reinforces this perspective by highlighting that adaptive outcomes emerge not merely from reactive responses but from the sustained capacity to transform structures, processes, and routines under uncertainty. ^17, 18^ The literature exemplifies practical applications in France, Vietnam, Canada, and Ghana, indicating that SMEs tailor reconfiguration and flexibility mechanisms to contextual realities, thereby enhancing adaptive resilience.

Despite these insights, persistent challenges remain. Empirical evidence is limited regarding the scalability of reconfiguration practices across SMEs with varying resource endowments, particularly in emerging economies where access to technology and skilled labour is constrained. ^87, 88^ Additionally, the interaction between strategic cognition and entrepreneurial experimentation in shaping long-term adaptability is underexplored, leaving gaps in understanding how SMEs institutionalise these practices systematically. Policy interventions can address these gaps by providing targeted support for resource recombination and flexibility. Governments and industry bodies could facilitate technology access through grants and subsidised equipment, promote skills development programmes tailored to flexible operations, and establish collaborative platforms for SMEs to share reconfiguration best practices. Incentivising incremental innovation and context-sensitive organisational restructuring can further strengthen adaptive capacity, ensuring that SMEs not only respond to immediate disruptions but also evolve strategically over time. ^86, 88^

### Strategic learning and knowledge integration in manufacturing SMEs: Theoretical alignment

The literature on strategic learning and knowledge integration in manufacturing SMEs aligns strongly with both Dynamic Capabilities Theory and Organisational Resilience Theory. Dynamic Capabilities Theory frames learning as a core mechanism through which firms strengthen their capacity to sense, seize, and reconfigure resources under uncertainty. ^85, 89^ Strategic thinking provides SMEs with a structured approach to reflective learning, enabling deliberate evaluation of past disruptions and the extraction of actionable insights. ^91, 94^ Entrepreneurial orientation complements this by fostering experimentation, trial-and-error initiatives, and innovation-driven knowledge creation, which expands the experiential base for future decision-making. ^74, 97^ Cross-functional integration and the establishment of external knowledge networks demonstrate how learning is operationalised to support coordinated responses across organisational domains, aligning internal capabilities with external opportunities and threats. ^98, 102^ Organisational Resilience Theory further underscores the role of continuous learning and knowledge integration as dynamic processes that enable firms not merely to recover from shocks but to adapt and transform over time. ^17, 18^ Collectively, these frameworks explain how SMEs embed adaptive resilience into strategic routines through multi-level, continuous learning processes.

Despite these theoretical linkages, the literature highlights several persistent gaps and challenges. Evidence remains limited on how SMEs in resource-constrained or institutionally diverse contexts systematically implement learning mechanisms that combine reflective thinking with experimental action. ^103, 104^ There is also a lack of empirical studies exploring how knowledge integration across geographically dispersed units can be standardised without undermining contextual adaptability, particularly in SMEs operating internationally. ^105, 106^ Policy interventions could address these gaps by promoting structured learning frameworks, supporting collaborative platforms for cross-functional and inter-organisational knowledge sharing, and incentivising innovation-led experimentation. Governments and industry associations can facilitate training programmes on knowledge management, provide access to shared research and technology platforms, and support international collaboration networks to enhance cross-context learning. Such measures would enable SMEs to harness strategic learning and knowledge integration effectively, thereby reinforcing adaptive resilience and sustaining competitive advantage in volatile manufacturing environments.

### Strategic renewal and long-term adaptability in manufacturing SMEs: Theoretical alignment

The literature on strategic renewal and long-term adaptability in manufacturing SMEs aligns closely with Dynamic Capabilities Theory and Organisational Resilience Theory, highlighting renewal as the apex of adaptive resilience. Dynamic Capabilities Theory positions resource reconfiguration, learning, and innovation as iterative processes through which firms sustain competitiveness under uncertainty. ^92, 107^ Strategic thinking enables SMEs to periodically reframe strategic intent, reassess value creation, and align short-term actions with long-term objectives, providing cognitive coherence across temporal horizons. ^109, 112^ Entrepreneurial orientation complements this by embedding continuous innovation and opportunity-driven behaviour into organisational routines, ensuring that experimentation and creativity are not reactive but integral to strategy. ^113, 115^ Organisational Resilience Theory further reinforces that resilience extends beyond recovery to sustained adaptability, emphasising the role of institutional embeddedness and proactive engagement with policy, cultural, and market structures. ^17, 18, 119, 121^ Together, these theoretical perspectives explain how SMEs integrate strategic cognition, entrepreneurial action, and contextual awareness to transform adaptive capabilities into long-term strategic renewal.

Despite these theoretical insights, the literature identifies persistent challenges and gaps. Empirical studies remain limited on the mechanisms through which SMEs balance short-term operational adaptations with long-term strategic vision, particularly in resource-constrained environments. ^116, 117^ There is also a scarcity of research examining how SMEs embed renewal processes across diverse institutional contexts while maintaining flexibility and innovation pipelines. ^120, 121^ Policy interventions could address these gaps by promoting supportive regulatory frameworks, innovation incentives, and skills development programmes. Governments and industry associations might provide guidance for scenario-based strategic planning, offer tax incentives for sustained R&D investment, and facilitate collaborative platforms for knowledge and technology exchange. Such measures would enhance SMEs’ capacity to institutionalise strategic renewal, maintain long-term adaptability, and strengthen resilience in dynamic and volatile global manufacturing landscapes.

### Policy implications for enhancing adaptive resilience in manufacturing SMEs

The literature review underscores that adaptive resilience in manufacturing SMEs is shaped by the integrated influence of strategic thinking and entrepreneurial orientation, manifested through environmental sensing, opportunity seizing, resource reconfiguration, strategic learning, and renewal. Policy interventions can therefore play a pivotal role in creating enabling environments that strengthen these capabilities. Governments and industrial regulators should prioritise policies that facilitate timely access to market intelligence, technological innovations, and sector-specific knowledge. For instance, supporting platforms for real-time industry data, digital innovation hubs, and cluster-based knowledge sharing can enhance SMEs’ strategic environmental sensing and anticipatory decision-making. Policies promoting research and development incentives, technology adoption grants, and collaborative initiatives between SMEs, universities, and research institutions can stimulate entrepreneurial experimentation and proactive opportunity seizing, reducing barriers to innovation and calculated risk-taking in dynamic manufacturing contexts.

In addition, institutional support mechanisms should encourage flexibility and resource reconfiguration within SMEs. Policymakers can offer targeted financial instruments, such as low-interest loans, tax incentives, or grants for upgrading production systems and adopting sustainable technologies, enabling firms to strategically recombine assets and experiment with new operational models. Labour market policies that encourage skills development, vocational training, and flexible workforce arrangements can further facilitate organisational adaptability and cross-functional collaboration. Moreover, long-term industrial policies that integrate sustainability, clean technology, and digitalisation objectives can reinforce strategic renewal processes, ensuring that SMEs’ short-term adaptive actions align with national development priorities. Across emerging and developed markets, these policy interventions create structural and institutional conditions that enhance the strategic and entrepreneurial capabilities of SMEs, promoting sustained adaptive resilience in volatile global manufacturing environments.

### Directions for future research on adaptive resilience in manufacturing SMEs

The review highlights the critical interplay between strategic thinking and entrepreneurial orientation in fostering adaptive resilience through sensing, seizing, reconfiguring, learning, and renewal capabilities. Future research could explore the contextual contingencies that influence this interplay, particularly in under-researched institutional settings such as sub-Saharan Africa, Southeast Asia, and Latin America. Comparative studies examining how SMEs across different regulatory, cultural, and technological environments develop and deploy dynamic capabilities would deepen understanding of contextual adaptation mechanisms and provide evidence for tailoring resilience strategies to specific institutional conditions.

Additionally, longitudinal research is needed to investigate how strategic thinking and entrepreneurial orientation interact over time to sustain adaptive resilience, especially under persistent volatility, technological disruption, and global supply chain shocks. Empirical studies could focus on measuring the impact of integrated dynamic capabilities on firm performance, innovation outcomes, and long-term strategic renewal, using multi-level data across organisational functions and networks. There is also scope to examine the role of digital technologies, data analytics, and Industry 4.0 tools in enhancing strategic environmental sensing and knowledge integration. Finally, future research could evaluate the effectiveness of specific policy interventions in strengthening SME resilience, providing actionable insights for governments and industry bodies seeking to support sustainable competitiveness in global manufacturing sectors.

## Conclusion

This review demonstrates that adaptive resilience in manufacturing SMEs emerges from the integrated application of strategic thinking and entrepreneurial orientation across multiple dimensions of dynamic capabilities. Strategic environmental sensing and opportunity anticipation enable firms to interpret complex, uncertain environments, identify weak signals, and anticipate shifts in markets, technology, and regulation. Opportunity seizing translates these insights into decisive action through strategic resource allocation, calculated risk-taking, rapid response, and alignment with institutional frameworks. Resource reconfiguration and organisational flexibility provide the mechanisms for continuous internal adaptation, allowing SMEs to recombine assets, experiment innovatively, and restructure organisational processes to sustain performance under volatility. Strategic learning and knowledge integration reinforce resilience by embedding reflective practices, cross-functional collaboration, and contextualised knowledge transfer, ensuring that lessons from experience inform future strategic choices. Finally, strategic renewal and long-term adaptability consolidate these capabilities, enabling SMEs to continuously evolve, maintain competitive relevance, and align short-term actions with long-term objectives.

Despite these insights, the review highlights persistent challenges. SMEs often face limited access to financial and technological resources, institutional constraints, and skills shortages, which hinder effective sensing, seizing, and reconfiguration. Additionally, uneven adoption of strategic thinking and entrepreneurial practices across firms leads to variability in resilience outcomes, while contextual complexities in emerging markets can impede the transfer and integration of knowledge. Market volatility and rapid technological change also exacerbate the difficulty of maintaining coherence between short-term adaptation and long-term strategic intent.

The findings therefore underscore that resilience in manufacturing SMEs is not merely about surviving disruption but about embedding proactive, forward-looking, and contextually informed capabilities into organisational routines. Strategic thinking provides the cognitive architecture for coherence, foresight, and alignment, while entrepreneurial orientation energises innovation, experimentation, and opportunity-driven action. Addressing the identified challenges requires targeted support, including access to finance, technology, and capability-building programs, as well as policies that reduce institutional constraints and encourage knowledge exchange. By fostering these conditions, SMEs can overcome resource and contextual limitations, strengthen adaptive capabilities, and sustain long-term competitiveness in volatile global manufacturing environments.

### Recommendations for policy and practice

Based on the findings of this review, actionable recommendations emerge for strengthening adaptive resilience in manufacturing SMEs through coordinated policy and practical interventions. Policymakers should prioritise initiatives that reduce structural barriers while facilitating access to finance, technology, and capacity building. National and regional governments, industrial development agencies, and economic planning bodies can design targeted support programmes such as innovation grants, subsidised technology adoption schemes, and strategic management training tailored to SME needs. For example, in Sweden, manufacturing SMEs have successfully aligned opportunity seizing with strong policy support for sustainability and innovation by leveraging government grants, green financing instruments, and collaborative innovation platforms, enabling firms to pursue advanced manufacturing and clean technology initiatives that reinforce resilience and competitive positioning. ^66– 68^ Such interventions create an enabling environment for proactive environmental sensing, resource reconfiguration, and strategic renewal, ensuring that SMEs can translate emerging opportunities into long-term adaptive success.

At the practice level, SME leaders should embed adaptive processes into organisational routines by fostering strategic thinking, entrepreneurial experimentation, and knowledge integration. This includes developing systematic environmental scanning mechanisms, cross-functional learning structures, and piloting programmes to encourage incremental innovation. Industrial associations, chambers of commerce, and innovation hubs are key stakeholders in supporting these initiatives by facilitating knowledge exchange, connecting SMEs with research institutions, and exposing firms to global market trends. A relevant example is found in German precision-engineering SMEs, which strategically interpreted regulatory and technological signals not merely as compliance costs but as indicators of future market demand, enabling early investment in energy-efficient machinery and digital production systems that positioned them ahead of competitors when sustainability standards became mandatory. ^28, 29^ Similarly, agribusiness SMEs in Chile used early climate and global commodity trend data to anticipate disruptions in raw material availability, diversifying supplier bases and investing in alternative inputs to sustain continuity in production and market supply. ^46^

Collaboration between policymakers, industry stakeholders, and SME leaders is essential to institutionalise these practices. Strategies should include public–private partnerships integrating financial support, technology transfer, and skills development, as well as regulatory reforms that encourage flexibility and innovation. Industrial clusters, export promotion agencies, and trade associations can also promote best-practice sharing and collective learning forums. By combining enabling policy frameworks with proactive organisational practices, manufacturing SMEs can overcome resource constraints, navigate institutional complexities, and sustain long-term adaptability. Ultimately, these recommendations highlight that resilience is a dynamic capability strengthened through coordinated action across multiple levels, allowing SMEs to harness uncertainty as strategic opportunity.

## Data Availability

No data are associated with this article.

## References

[1] Epede MB Wang D : Global value chain linkages: An integrative review of the opportunities and challenges for SMEs in developing countries. *Int. Bus. Rev.* 2022 Oct 1;31(5):101993. 10.1016/j.ibusrev.2022.101993

[2] Cragg T McNamara T Descubes I : Manufacturing SMEs, network governance and global supply chains. *J. Small Bus. Enterp. Dev.* 2020 Feb 4;27(1):130–147. 10.1108/JSBED-10-2019-0334

[3] Munna MS : Global Disruptions and SME Resilience: A Framework for Strategic Adaptation. 2025.

[4] Tariq MU : Innovative strategies for enhancing SME competitiveness in emerging economies. *Models, Strategies, and Tools for Competitive SMEs.* IGI Global;2025; pp.151–172.

[5] Conz E Magnani G : A dynamic perspective on the resilience of firms: A systematic literature review and a framework for future research. *Eur. Manag. J.* 2020 Jun 1;38(3):400–412. 10.1016/j.emj.2019.12.004

[6] Kokogho E Odio PE Ogunsola OY : Conceptual analysis of strategic historical perspectives: Informing better decision making and planning for SMEs. *Journal of Strategic Management Research.* 2024 Nov;3(3):108–119. 10.54660/IJMOR.2024.3.6.108-119

[7] Azam SF Ling CP Karim KS : The Influence of Dynamic Capabilities and Strategic Orientation of SME Leaders. *International Journal on Management Education and Emerging Technology (IJMEET).* 2024 Dec 23;2(4):1–9.

[8] Corrêa VS Queiroz MM Cruz MA : Entrepreneurial orientation far beyond opportunity: the influence of the necessity for innovativeness, proactiveness and risk-taking. *Int. J. Entrep. Behav. Res.* 2022 May 10;28(4):952–979. 10.1108/IJEBR-06-2021-0518

[9] AlQershi N : Strategic thinking, strategic planning, strategic innovation and the performance of SMEs: The mediating role of human capital. *Management Science and Education Innovations.* 2024 Dec 17;1(19).

[10] Steen R Haug OJ Patriarca R : Business continuity and resilience management: A conceptual framework. *J. Conting. Crisis Manag.* 2024 Mar;32(1):e12501. 10.1111/1468-5973.12501

[11] Joulal N Messaoudi A : From Disruption to Continuity: Understanding Organizational Resilience Strategies in the Face of Technological Change. *Sch. J. Econ. Bus. Manag.* 2024 Sep;11:275–282. 10.36347/sjebm.2024.v11i09.005

[12] Zighan S Abualqumboz M Dwaikat N : The role of entrepreneurial orientation in developing SMEs resilience capabilities throughout COVID-19. *Int. J. Entrep. Innov.* 2022 Nov;23(4):227–239. 10.1177/14657503211046849

[13] Kwiotkowska A : The relationship between entrepreneurial orientation and organizational resilience in the digital context. *Organisation & Management.* 2022 Dec 15;2023:473–488. 10.29119/1641-3466.2022.166.30

[14] Teece DJ Pisano G Shuen A : Dynamic capabilities and strategic management. *Strateg. Manag. J.* 1997;18(7):509–533.

[15] Kurtmollaiev S : Dynamic capabilities and where to find them. *J. Manag. Inq.* 2020 Jan;29(1):3–16. 10.1177/1056492617730126

[16] Posen HE Ross JM Wu B : Reconceptualizing imitation: Implications for dynamic capabilities, innovation, and competitive advantage. *Acad. Manag. Ann.* 2023 Jan;17(1):74–112. 10.5465/annals.2021.0044

[17] Lengnick-Hall CA Beck TE Lengnick-Hall ML : Developing a capacity for organizational resilience through strategic human resource management. *Hum. Resour. Manag. Rev.* 2011;21(3):243–255.

[18] Butler T Brooks R : Achieving operational resilience in the financial industry: Insights from complex adaptive systems theory and implications for risk management. *Journal of Risk Management in Financial Institutions.* 2021 Sep 1;14(4):395–407. 10.69554/OUQJ2151

[19] Hamzah MI : Market, Entrepreneurial Orientations, Crisis Adaptiveness, and Performance of SMEs: A Moderated Mediation Analysis. *SAGE Open.* 2025 Oct;15(4):21582440251390323. 10.1177/21582440251390323

[20] Markarian S : *A Delphi Study on Strategic Behaviors From the Adaptive Leadership Framework Used by Successful CXOs for Business Agility in a Volatility, Uncertainty, Complexity, and Ambiguity Environment.* University of Massachusetts Global; Doctoral dissertation.

[21] Udoboho ME : Manufacturing Strategy Implementation Conceptualization: Challenges and Benefits in the Volatile, Uncertain, Complex and Ambiguous Business Environment. *Uncertain, Complex and Ambiguous Business Environment.* November 9, 2021. 2021 Nov 9. 10.2139/ssrn.3959821

[22] Kuzmanov I : The psychology of strategic communication and decision-making: Analytical acumen and cognitive agility in complex world. *Journal of Novel Research and Innovative Development.* 2025 Jan;3(1):a421–a437. 10.56975/jnrid.v3i6.701485

[23] Kamaldeen O : A Systemic Approach to Strategic Planning: Navigating Complexity with Clarity. 2024 Apr 15.

[24] Kalita A Chepurenko A : Competitiveness of small and medium businesses and competitive pressure in the manufacturing industry. *Форсайт.* 2020;14(2 (eng)):36–50. 10.17323/2500-2597.2020.2.36.50

[25] Lee Y *Evolution of Strategic Foresight and Policy Making: A Comparative Evaluation of British and American Strategies for National Emergency Crises.* United Kingdom: University of London, University College London;2025. Doctoral dissertation.

[26] Burtscher J Leipziger M Kanbach DK : Pathways to twin transformation in SMEs: the role of innovation ecosystems. *Eur. J. Innov. Manag.* 2025 Sep 9;1–26. 10.1108/EJIM-11-2024-1382

[27] Zall M Leutheuser V Müller JM : Digital manufacturing in SMEs: Green, efficient and socially responsible?. *Procedia Computer Science.* 2025 Jan 1;253:551–560. 10.1016/j.procs.2025.01.117

[28] Beier G Matthess M Guan T : Impact of Industry 4.0 on corporate environmental sustainability: Comparing practitioners’ perceptions from China, Brazil and Germany. *Sustainable Prod. Consumption.* 2022 May 1;31:287–300. 10.1016/j.spc.2022.02.017

[29] König W Löbbe S Büttner S : Establishing energy efficiency—drivers for energy efficiency in German manufacturing small-and medium-sized enterprises. *Energies.* 2020 Oct 2;13(19):5144. 10.3390/en13195144

[30] Amanah AA Hussein SA Bannay DF : Role of proactive behavior in entrepreneurial alertness: A mediating role of dynamic capabilities. *Probl. Perspect. Manag.* 2022;20(4):127.

[31] Makhloufi L Laghouag AA Ali Sahli A : Impact of entrepreneurial orientation on innovation capability: The mediating role of absorptive capability and organizational learning capabilities. *Sustainability.* 2021 May 12;13(10):5399. 10.3390/su13105399

[32] KONG WS : Entrepreneurial orientation in family firms: The role of intergenerational dynamics, firm size, and external working experience. 2025.

[33] Mittal S Khan MA Purohit JK : A smart manufacturing adoption framework for SMEs. *Int. J. Prod. Res.* 2020 Mar 3;58(5):1555–1573. 10.1080/00207543.2019.1661540

[34] Park C McQuaid R Mawson S : Key factors influencing the sustained growth of high-tech SMEs in South Korea: the perspectives of founder owner-managers. *Int. J. Entrep. Behav. Res.* 2023 Nov 29;29(9/10):2135–2156. 10.1108/IJEBR-12-2022-1077

[35] Lee JW : Analysis of technology-related innovation characteristics affecting the survival period of SMEs: Focused on the manufacturing industry of Korea. *Technol. Soc.* 2021 Nov 1;67:101742. 10.1016/j.techsoc.2021.101742

[36] Samson-Onuorah CI Bakare FA : Dynamic Strategic Foresight Using Predictive Business Analytics: Modeling Competitive Advantage Under Volatile Market and Technological Conditions. *International Journal of Research Publication and Reviews.* May 2025;6(5):1044–1060. 10.55248/gengpi.6.0525.1627

[37] Augusto JC : Contexts and context-awareness revisited from an intelligent environments perspective. *Appl. Artif. Intell.* 2022 Dec 31;36(1):2008644. 10.1080/08839514.2021.2008644

[38] Briggs ET Okon GU Ebong TI : Strategic Entrepreneurial Approaches and Their Influence on the Competitive Posture of Small-Scale Enterprises in South-South Nigeria. *Frontiers in Business Innovations and Management.* 2025 Jun 18;02(06):18–29. 10.64917/fbim-009

[39] Ibidunni AS Kolawole AI Olokundun MA : Knowledge transfer and innovation performance of small and medium enterprises (SMEs): An informal economy analysis. *Heliyon.* 2020 Aug 1;6(8):e04740. 10.1016/j.heliyon.2020.e04740 32885077 PMC7452491

[40] Mukhopadhyay U : Efficacy of cluster development initiatives in India: A study of zari embroidery clusters. *Int. J. Technol. Manag. Sustain. Dev.* 2025 Oct 1;24(3):291–314. 10.1386/tmsd_00109_1

[41] Kush JC : Integrating sensor technologies with conversational AI: enhancing context-sensitive interaction through real-time data fusion. *Sensors.* 2025 Jan 4;25(1):249. 10.3390/s25010249 39797040 PMC11723437

[42] Vettorello M : *Operationalising anticipatory systems: Navigating uncertainty and ambiguity to drive innovation at the fuzzy front end.* Swinburne;2022. Doctoral dissertation.

[43] Carter W : Financial Forecasting and Cognitive Biases: A Theoretical Examination of Framing Effects and Predictive Accuracy. 2025.

[44] Karami A Gorzynski RA : Connection to nature and sustainability in small-and medium-sized environmental organizations: A dynamic strategic thinking approach. *Bus. Strateg. Environ.* 2022 Jan;31(1):371–389. 10.1002/bse.2898

[45] Cheng P Wu S Xiao J : Exploring the impact of entrepreneurial orientation and market orientation on entrepreneurial performance in the context of environmental uncertainty. *Sci. Rep.* 2025 Jan 14;15(1):1913. 10.1038/s41598-025-86344-w 39814997 PMC11736034

[46] Rodriguez Cardenas N : Harvesting Innovation: Navigating Enablers and Challenges for Chilean Startups on the Path to Sustainable Food Ecosystems. 2024. (Master's thesis).

[47] Downs J : *Strategic thinking: an exploration.* RMIT University;2024. Doctoral dissertation.

[48] Mwanza PM Dar JA : Role of dynamic capabilities in public sector performance: a strategic management perspective. *International Journal of Research in Business & Social Science.* 2025 Jun 1;14(4):19–31. 10.20525/ijrbs.v14i4.4122

[49] Sapian DS : *SME strategic regeneration: Building a holistic framework for decision-making in managing growth uncertainties and market volatilities for SMEs in Malaysia.* United Kingdom: University of Wales Trinity Saint David;2022.

[50] Ooi SK Memon KR : Addressing Resource Scarcity: The Role of Responsible Innovation and Resilience in SMEs' Competitive Advantage and Sustainability Performance. *Corp. Soc. Responsib. Environ. Manag.* 2025;32:5734–5746. 10.1002/csr.70005

[51] Indrawati H Caska S : Barriers to technological innovations of SMEs: how to solve them?. *International Journal of Innovation Science.* 2020 Dec 9;12(5):545–564. 10.1108/IJIS-04-2020-0049

[52] Sunoto SP Daryanto E : The Role of Strategic Leadership in Building Organizational Resilience. *International Journal of Integrative Sciences.* 2025 Mar 24;4(3):517–532. 10.55927/ijis.v4i3.89

[53] Duić N Hendrickson C Yubo LI : Navigating the Future: A Report on the Current State and Future Path of Energy Transition in the Transport Sector. *Godišnjak Akademije tehničkih znanosti Hrvatske.* 2024;2024(1):193–249.

[54] Georgiev S Ohtaki S : Critical success factors for TQM implementation among manufacturing SMEs: Evidence from Japan. *BIJ.* 2020 Mar 21;27(2):473–498. 10.1108/BIJ-01-2019-0037

[55] Putniņš TJ Sauka A : Why does entrepreneurial orientation affect company performance?. *Strateg. Entrep. J.* 2020 Dec;14(4):711–735. 10.1002/sej.1325

[56] Yang J Johansson DJ : Adapting to uncertainty: Modeling adaptive investment decisions in the electricity system. *Appl. Energy.* 2024 Mar 15;358:122603. 10.1016/j.apenergy.2023.122603

[57] Khelil N Perrigot R Watson A : A processual understanding of fear of failure: Insights from small-business entrepreneurs affected by a crisis. *J. Small Bus. Manag.* 2025 Oct 3;1–43. 10.1080/00472778.2025.2544846

[58] Benincasa A : Optimizing decarbonization pathways for Italy's hard-to-abate industries: a high-detail spatial-temporal and technological modeling approach. 2024.

[59] Ahmad J : Strategic Planning: Navigating Uncertainty in Business Management. *Journal of Management & Social Science.* 2024 Mar 31;1(02):33–46.

[60] Brown S Bessant J : The manufacturing strategy-capabilities links in mass customisation and agile manufacturing–an exploratory study. *Int. J. Oper. Prod. Manag.* 2003 Jul 1;23(7):707–730. 10.1108/01443570310481522

[61] Vettorello M Eisenbart B Ranscombe C : The innovation system roadmap: A novel approach to instil futures-oriented reasoning in strategic decision making. *Creat. Innov. Manag.* 2022 Mar;31(1):5–18. 10.1111/caim.12472

[62] Parnell JA Long Z Lester D : Competitive strategy, capabilities and uncertainty in small and medium sized enterprises (SMEs) in China and the United States. *Manag. Decis.* 2015 Mar 16;53(2):402–431. 10.1108/MD-04-2014-0222

[63] Morgan T Anokhin SA : The joint impact of entrepreneurial orientation and market orientation in new product development: Studying firm and environmental contingencies. *J. Bus. Res.* 2020 May 1;113:129–138. 10.1016/j.jbusres.2019.06.019

[64] Gatignon A Capron L : The firm as an architect of polycentric governance: Building open institutional infrastructure in emerging markets. *Strateg. Manag. J.* 2023 Jan;44(1):48–85. 10.1002/smj.3124

[65] Sawaean F Ali K : The impact of entrepreneurial leadership and learning orientation on organizational performance of SMEs: The mediating role of innovation capacity. *Manag. Sci. Lett.* 2020;10(2):369–380.

[66] Tsvetkova D Bengtsson E Durst S : Maintaining sustainable practices in SMEs: Insights from Sweden. *Sustainability.* 2020 Dec 8;12(24):10242. 10.3390/su122410242

[67] Burman F Österberg C : Unlocking growth for SMEs in the Swedish public sector: Strategic approaches for navigating challenges in the Swedish public sector as a small player. 2024.

[68] Adenekan TK : Green Innovation in the Age of Financial Development and Government R&D Subsidies. 2024.

[69] Hsu HY Chang TS : Institutionalizing ESG in SMEs: Insights From Transformational Leadership and Institutional Theory. *Business Strategy & Development.* 2026 Mar;9(1):e70264. 10.1002/bsd2.70264

[70] Patrício V Costa RL Carvalho H : Investigation model of sensing, seizing and reconfiguring capabilities. *International Journal of Value Chain Management.* 2022;13(4):395–421.

[71] Thomas GH Douglas EJ : Resource reconfiguration by surviving SMEs in a disrupted industry. *J. Small Bus. Manag.* 2024 Jan 2;62(1):140–174. 10.1080/00472778.2021.2009489

[72] Chaturvedi T Prescott JE : Resource reconfiguration during technological change. *Strategy Science.* 2022 Sep;7(3):240–265. 10.1287/stsc.2021.0151

[73] Lee JM Narula R Hillemann J : Unraveling asset recombination through the lens of firm-specific advantages: A dynamic capabilities perspective. *J. World Bus.* 2021 Feb 1;56(2):101193. 10.1016/j.jwb.2021.101193

[74] Gomes G Seman LO Berndt AC : The role of entrepreneurial orientation, organizational learning capability and service innovation in organizational performance. *Revista de gestão.* 2022 Jan 13;29(1):39–54. 10.1108/REGE-11-2020-0103

[75] Elfghi O Iyiola K Alzubi AB : Improvisation and New Venture Performance: Unpacking the Roles of Entrepreneurial Self-Efficacy and Learning Orientation. *Sustainability.* 2026 Jan 18;18(2):975. 10.3390/su18020975

[76] Ngoc NB Ngoc BD Manh HT : Forest and Forest Products Sector Development Based on Hardwood Plantations in Vietnam. *8th–International Scientific Conference on Hardwood Processing.* p.10.

[77] Polyanskaya O Shaitarova O Tereshenko S : Problems and prospects for the development of the furniture industry in Vietnam. *IOP Conference Series: Earth and Environmental Science.* IOP Publishing;2020 Oct 1; Vol.574(1): p.012067.

[78] Van Hoang D Thi Hien N Van Thang H : Digital capabilities and sustainable competitive advantages: the case of emerging market manufacturing SMEs. *SAGE Open.* 2025 Apr;15(2):21582440251329967. 10.1177/21582440251329967

[79] Wang G Li X Zhou J : The influence of entrepreneurial team’s cognitive adaptability on its risk decision making. *Ind. Manag. Data Syst.* 2020 Jan 22;120(2):329–349. 10.1108/IMDS-03-2019-0178

[80] Dhir S : Strategic Management: Reflecting Strategic Flexibility, Innovation and Entrepreneurship. *Springer Nature.* 2024 Sep 25.

[81] Ali MH Suleiman N Khalid N : Supply chain resilience reactive strategies for food SMEs in coping to COVID-19 crisis. *Trends Food Sci. Technol.* 2021 Mar 1;109:94–102. 10.1016/j.tifs.2021.01.021 34728899 PMC8554876

[82] Roberts MJ Muralidharan E : Internationalization of service SMEs: perspectives from Canadian SMEs internationalizing in Asia. *Glob. Bus. Rev.* 2022 Aug;23(4):890–910. 10.1177/0972150919887250

[83] Taherizadeh A Beaudry C : An emergent grounded theory of AI-driven digital transformation: Canadian SMEs’ perspectives. *Ind. Innov.* 2023 Oct 21;30(9):1244–1273. 10.1080/13662716.2023.2242285

[84] Alateeg S Alhammadi A : The impact of organizational culture on organizational innovation with mediation role of strategic leadership in Saudi Arabia. *Journal of Statistics Applications & Probability.* 2024;13(2):843–858.

[85] Quansah E Hartz DE Salipante P : Adaptive practices in SMEs: leveraging dynamic capabilities for strategic adaptation. *J. Small Bus. Enterp. Dev.* 2022 Oct 18;29(7):1130–1148.

[86] Kahpi HS Wulandari SS Atichasari AS : Analysis of the relationship between innovative leadership and market orientation on the sustainability of technopreneurship: the mediating role of external regulatory compliance: an empirical evidence in Indonesia. *Environ. Dev. Sustain.* 2024 May 31;1–42. 10.1007/s10668-024-05072-9

[87] Kwaku AR Hui X Samuel A : Unlocking Opportunities in Agro-Industrialization and Agro-Processing for Inclusive Growth in Ghana. *Cognizance Journal of Multidisciplinary Studies.* 2024;4(1):357–395.

[88] Avudufu FY : *Drivers of Small-Scale agribusiness performance in Ghana: Evidence from the Nkoranza South district.* Central University of Technology;2022. Doctoral dissertation.

[89] Zungu Z Laryea S : Leveraging Sensing as a Dynamic Capability: A Strategy-as-Practice Approach to Enhancing Organizational Resilience in Construction Firms. *CIB Conferences.* 2025; Vol.1(1): p.317.

[90] Suherlan S Purnama Y : STRATEGIC ANALYSIS OF THE EXPERIENTIAL LEARNING APPROACH IN DEVELOPING YOUTH ENTREPRENEURIAL CAPABILITIES: A PERSPECTIVE ON PEDAGOGICAL INNOVATION AND CHARACTER TRANSFORMATION. *Technopreneurship and Educational Development Review (TENDER).* 2025 Jul 1;2(2):161–169.

[91] Kim L Imjai N Kaewjomnong A : Does experiential learning matter to strategic intuition skills of MBA students? Implications of diagnostic capabilities and critical thinking skills. *The International Journal of Management Education.* 2025 Jul 1;23(2):101138. 10.1016/j.ijme.2025.101138

[92] Carayannis EG Dumitrescu R Falkowski T : Enhancing SME resilience through artificial intelligence and strategic foresight: A framework for sustainable competitiveness. *Technol. Soc.* 2025 Jun 1;81:102835. 10.1016/j.techsoc.2025.102835

[93] Manville G Papadopoulos T Garengo P : Twenty-first century supply chain management: a multiple case study analysis within the UK aerospace industry. *Total Qual. Manag. Bus. Excell.* 2021 May 19;32(7-8):869–885. 10.1080/14783363.2019.1642101

[94] Luna Andrade JJ : Analysis of the evolution of aerospace manufacturing ecosystems. 2020.

[95] Andersen TC Aagaard A Magnusson M : Exploring business model innovation in SMEs in a digital context: Organizing search behaviours, experimentation and decision-making. *Creat. Innov. Manag.* 2022 Mar;31(1):19–34. 10.1111/caim.12474

[96] Gungor A Bamforth CJ Dalrymple J : An investigation of factors affecting Australian small and medium enterprise performance during The Pandemic. *J. Innov. Entrep.* 2025 Jul 1;14(1):80. 10.1186/s13731-025-00532-6

[97] Ortega-Del-Rosario MD Caballero R Domínguez MA : AI-Enhanced Manufacturing in Latin America: Opportunities, Challenges, Applications, and Regulatory Policy Frameworks for Intelligent Production Systems. *Appl. Sci.* 2025 Oct 15;15(20). 2076-3417.

[98] Zahra SA Neubaum DO Hayton J : What do we know about knowledge integration: Fusing micro-and macro-organizational perspectives. *Acad. Manag. Ann.* 2020 Jan;14(1):160–194. 10.5465/annals.2017.0093

[99] Li X Peng Z Li K : Impact of boundary-spanning search on firm innovation performance: a strategic knowledge integration perspective. *J. Knowl. Manag.* 2024 Nov 19;28(10):3075–3103. 10.1108/JKM-07-2023-0575

[100] Zieba K : Knowledge management and resilience in SMEs sector. *European Conference on Knowledge Management.* Academic Conferences International Limited;2024 Sep 1; pp.944–950.

[101] Leogrande A Costantiello A Laureti L : The Export of Medium and High-Tech Products Manufactured in Europe. *Journal of Applied Economic Sciences.* 2022 Sep 1;17(3).

[102] Rošulj D Petrović DČ Arsić SM : Knowledge management in Serbian SMEs: key factors of influence on internal and external business performances. *Sustainability.* 2024 Jan 17;16(2):797. 10.3390/su16020797

[103] Arslan K Savaş G Kılınç AÇ : Enhancing teacher innovative practices through principal learning-centred leadership and teacher knowledge sharing. *Br. Educ. Res. J.* 2025 Dec 19. 10.1002/berj.70067

[104] Mohammad RA Alahmari AM Faqih RH : Linking strategic intelligence, strategic leadership, strategic planning, and strategic thinking and business performance: the moderating effect of strategic flexibility. *Discov. Sustain.* 2024 Nov 26;5(1):434. 10.1007/s43621-024-00670-z

[105] Arghashi V Okumuş A : Country-of-origin image; SMEs and emerging economies–evidence from a case study of manufacturing SMEs from Turkey. *Journal of Islamic Marketing.* 2022 Mar 7;13(4):956–974. 10.1108/JIMA-04-2020-0106

[106] Filiz U : *A comparative analysis on IHRM policies and practices at local subsidiaries of MNCs in transitional and emerging environment: The case of Hungary and Turkey.* Magyar Agrár-és Élettudományi Egyetem;2022. Doctoral dissertation.

[107] Goronga P Matongo M Deredzai M : Adapting to the Unpredictable and Uncertain Academic Environments: Strategies for Building Resilience. *Building Organizational Capacity and Strategic Management in Academia.* IGI Global;2025; pp.35–66.

[108] Scharunge J Puth AC : Managing Strategic Entrepreneurship in SMEs: Top Managers Engaging in Advantage-Seeking and Opportunity-Seeking. 2020.

[109] Mukherjee M Ramirez R Cuthbertson R : Strategic reframing as a multi-level process enabled with scenario research. *Long Range Plan.* 2020 Oct 1;53(5):101933. 10.1016/j.lrp.2019.101933

[110] Dahlquist S Lehnert K : Strategic Orientation: Market changer or market defender?. *J. Macromark.* 2023 Mar;43(1):5–16. 10.1177/02761467221134024

[111] Borner S : internationalization Competitiveness and of industry: the case of Switzerland. *The Competitiveness of European Industry.* 2023 Feb 28;64. 10.4324/9781003369820-5

[112] Agu EE Nwabekee US Ijomah TI : The role of strategic business leadership in driving product marketing success: Insights from emerging markets. *International Journal of Frontline Research in Science and Technology.* 2024;3(02):001–018.

[113] Abdulsamad A Ateeq A Al-Zubaidi R : Entrepreneurial Orientation and Innovation Capabilities as Drivers of Sustainable Innovation Performance: A Conceptual Framework for SMEs. *Tech Fusion in Business and Society.* 2025;845–858. 10.1007/978-3-031-84636-6_76

[114] Oiku PO : Innovation and organisational resilience among small and medium-sized enterprises in Lagos State. *International Journal of Business and Technopreneurship (IJBT).* 2024 Feb 27;14(1):35–48. 10.58915/ijbt.v14i1.298

[115] Heilala J Helaakoski H Kuivanen R Kääriäinen J : A review of digitalisation in the Finnish manufacturing SME companies. 2020.

[116] Carreño AM : *Strategic alignment in program management: A framework for sustainable business transformation.* Institute for Change Leadership and Business Transformation;2024 Sep 2;13922003.

[117] Juurinen E : Identifying the Dimensions of Dynamic Capabilities and Entrepreneurial Orientation for Success and Failure in Organizational Adaptation to Changing Environments.

[118] Ali I Aboelmaged MG : Implementation of supply chain 4.0 in the food and beverage industry: perceived drivers and barriers. *Int. J. Product. Perform. Manag.* 2022 Apr 8;71(4):1426–1443. 10.1108/IJPPM-07-2020-0393

[119] Nowak V Raffaelli P : The interconnected influences of institutional and social embeddedness on processes of social innovation: A Polanyian perspective. *Entrep. Reg. Dev.* 2022 Mar 15;34(3-4):319–342. 10.1080/08985626.2022.2049376

[120] Habibullah M Kamal A : Environmental dynamism and strategic performance in small and medium enterprises. *Journal of Energy and Environmental Policy Options.* 2024 Sep 1;7(3):35–42.

[121] Twiringiyimana R : *Fostering knowledge space actor interactions in an emerging place-based innovation ecosystem: The case of Kigali Innovation City.* UCL (University College London);2025. Doctoral dissertation.

