# Supplementary material for: Strategic Thinking and Entrepreneurial Orientation as Drivers of Adaptive Resilience in Global Manufacturing SMEs: A Narrative Review of Dynamic Capabilities and Cross-Context Variations
Source: F1000Res. 2026 Mar 3;15:344. [Version 1] doi: 10.12688/f1000research.178316.1 (PMC13087567; doi:10.12688/f1000research.178316.1)
Supplement: Peer review history

# Reviewer report: Reviewer response for version 1

**DOI:** 10.5256/f1000research.196687.r464769

**Published:** 2026-04-16

**Authors:** Galvarino Casanueva Yáñez

**Affiliations:** 1 Ingeniería Comercial, Universidad de Las Americas - Campus Providencia (Ringgold ID: 518134), Santiago, Santiago Metropolitan Region, Chile

Good work overall. It should be noted that the topic is timely and of high academic and practical value. With a more precise operational definition of concepts, a more explicit theoretical framework, and a clear methodological protocol, the work could become a significant contribution that links dynamic capabilities theory with organizational resilience in global manufacturing SMEs, contributing to both academia and strategic management in contexts of uncertainty. For now, its indexing is viable, noting that deeper studies on the topic can be developed.

Is the review written in accessible language?

Yes

Are all factual statements correct and adequately supported by citations?

Yes

Are the conclusions drawn appropriate in the context of the current research literature?

Yes

Is the topic of the review discussed comprehensively in the context of the current literature?

Yes

Reviewer Expertise:

My area of ​​expertise is the administration and study of SMEs. I have written books on the subject, including "Manual for Entrepreneurs" and "Ultraproductive Entrepreneurs (Using AI for Management)."

I confirm that I have read this submission and believe that I have an appropriate level of expertise to confirm that it is of an acceptable scientific standard.

# Reviewer report: Reviewer response for version 1

**DOI:** 10.5256/f1000research.196687.r472445

**Published:** 2026-04-13

**Authors:** Ashraf Mishrif

**Affiliations:** 1 Sultan Qaboos University (Ringgold ID: 37611), Muscat, Oman

This paper provides a detailed review of how strategic thinking and entrepreneurial orientation interact to foster adaptive resilience in global manufacturing SMEs through dynamic capabilities and cross-context variations. It also seeks to advance strategic management and entrepreneurship theory by positioning adaptive resilience as a dynamic, capability-based outcome of strategic cognition and entrepreneurial behaviour.

 Conceptually, the paper bases its analysis on the dynamic capabilities’ theory, indicating that firm competitiveness depends not only on the possession of valuable resources but on the capacity to integrate, build, and reconfigure internal and external competence in response to rapid environmental changes. This analytical base has helped in defining the conceptual framework and providing meaningful explanations of the concepts of strategic thinking, entrepreneurial orientation, adaptive resilience, dynamic capabilities, and strategic cognition.

 Methodologically, the paper adopts a systematic qualitative review approach to collect data on adaptive resilience in manufacturing SMEs from peer-reviewed and grey literature between 2020 and 2025. This approach proved useful in collecting and analyzing data, using thematic synthesis to allow for the identification of recurring patterns, mechanisms and contextual factors across the reviewed studies. It uses Nvivo software that is useful to code, categorize and visualize the thematic relationships, facilitating systematic comparison across diverse studies and regional contexts.  

 In terms of its analysis and discussion, the paper is praised for its critical review of key strategic capabilities, including strategic environment sensing and opportunity anticipation, opportunity seizing through commitment and entrepreneurial action, resources reconfiguration and organizational flexibility, strategic learning and knowledge integration, and strategic renewal and long-term adaptability. While thoroughly analyzing these capabilities, the paper underlines significant research gap in literature, including the need to explore the contextual contingences that influence the interplay between strategic thinking and entrepreneurial orientation in fostering adaptive resilience, as well as measuring the impact of integrated dynamic capabilities on firm performance and innovation outcomes using multi-level data across organizational functions and networks.   

 Overall, the paper is well-argued and analyzed, offering some practical and policy implications and calling for increased collaboration between policymakers, industry stakeholders and company executives to institutionalize practices and integrate financial support, technology transfer, and skills development in the firm’s organizational structure and practices.

Is the review written in accessible language?

Yes

Are all factual statements correct and adequately supported by citations?

Yes

Are the conclusions drawn appropriate in the context of the current research literature?

Yes

Is the topic of the review discussed comprehensively in the context of the current literature?

Yes

Reviewer Expertise:

Economics

I confirm that I have read this submission and believe that I have an appropriate level of expertise to confirm that it is of an acceptable scientific standard.
